# Unraveling the impact of marine heatwaves on the Eukaryome of the emblematic Mediterranean red coral *Corallium rubrum*

**DOI:** 10.1093/ismeco/ycaf035

**Published:** 2025-02-21

**Authors:** Camille Prioux, Christine Ferrier-Pagès, Javier del Campo, Laure Guillou, Tristan Estaque, Denis Allemand, Romie Tignat-Perrier

**Affiliations:** Sorbonne Université Collège Doctoral, Science de l'environnement d'Ile de France, 75006, Paris, France; Unité de Recherche sur la Biologie des Coraux Précieux CSM - CHANEL, Centre Scientifique de Monaco, Monaco, 98000, Principality of Monaco; Centre Scientifique de Monaco, Quai Antoine 1er, Monaco, 98000, Principality of Monaco; Centre Scientifique de Monaco, Quai Antoine 1er, Monaco, 98000, Principality of Monaco; Department of Marine Biology and Ecology, Rosenstiel School of Marine Atmospheric and Earth Science, University of Miami, Miami, FL, United States; Biodiversitat, Institut de Biologia Evolutiva (CSIC-Universitat Pompeu Fabra), Barcelona, Spain; Sorbonne Université CNRS, UMR7144 Adaptation et Diversité en Milieu Marin, Ecology of Marine Plankton (ECOMAP), Station Biologique de Roscoff SBR, Roscoff, France; Septentrion Environnement, Campus Nature Provence, Traverse Parangon, Marseille, France; Centre Scientifique de Monaco, Quai Antoine 1er, Monaco, 98000, Principality of Monaco; Unité de Recherche sur la Biologie des Coraux Précieux CSM - CHANEL, Centre Scientifique de Monaco, Monaco, 98000, Principality of Monaco; Centre Scientifique de Monaco, Quai Antoine 1er, Monaco, 98000, Principality of Monaco

**Keywords:** microeukaryotic communities, climate change, Dino-Group I, labyrinthulomycetes, apicomplexa, *18S rRNA* gene sequencing, mass mortality events, octocorals, marine animal forests, temperate corals

## Abstract

Global warming is intensifying heatwaves worldwide, leading to more frequent and severe temperature extremes. This study investigates the impact of the unprecedented 2022 Mediterranean heatwaves on the coral eukaryome, which has received little attention despite its known importance to coral holobiont functioning. Fifty-six colonies of the iconic red coral *Corallium rubrum* from the Mediterranean Sea were collected at different sites, depths, and health states. The microeukaryotic communities were analyzed using an *18S rRNA* gene metabarcoding approach. Primers were designed to reduce amplification of the *18S rRNA* gene sequences of the red coral while being universal for amplification of microeukaryotes. Our results showed that the red coral eukaryome was dominated by Dino-Group I, Licnophoridae, and Labyrinthulomycetes in the control sites that were not affected by the heat waves. In the heat-affected colonies, the composition of the coral eukaryome changed, with the relative abundances of Ephelotidae, Exobasidiomycetes, Corallicolidae, Labyrinthulomycetes, and/or the epibionts Phaeophyceae increasing depending on the intensity of heat stress experienced by the colonies. It was thus possible to link colony health to changes in the eukaryome. Finally, we illustrated putative interactions (competition, predator–prey relationship, and parasitism) occurring within *C. rubrum* eukaryome that could explain the compositional changes observed in the microeukaryotic communities under heat stress. Our findings improve our understanding of the ecological effects of heatwaves on marine ecosystems.

## Introduction

Unicellular microeukaryotes, which include phototrophs, decomposers, predators, and symbionts, are fundamental components of marine and terrestrial ecosystems. As microeukaryotes have varied functional roles, they contribute to the length and connectivity of food webs across trophic levels [1–5]. In marine environments, ciliates regulate bacterial communities [6], fungi degrade organic matter [7, 8], and diatoms and dinoflagellates play a central role in the global carbon cycle [9, 10]. Microeukaryotes are also a key component of the microbiome in animals, including corals. Together with the host and its other associated microbial communities, they form a holobiont that functions as an integrated unit [11]. In corals, the microeukaryotic community, or eukaryome, likely plays important roles in processes such as nutrient cycling and immune responses [11]. However, despite its potential significance, the coral eukaryome remains poorly understood, highlighting the need for more research [4].

Symbiotic dinoflagellates from the Symbiodiniaceae family are the most well-studied microeukaryotic partners of corals, providing their host with photosynthates and meeting the majority of its energy requirements [12, 13]. Many other microeukaryotes are associated with corals, although our understanding of these associations is limited. Marine alveolates related to Syndiniales dinoflagellates, particularly the Dino-Group I subgroup, have been shown to dominate the eukaryome of the tropical hexacoral *Pocillopora damicornis* [14] and the heat-resistant colonies of the Mediterranean octocoral *Paramuricea clavata* [15]. In *P. damicornis*, their occurrence in healthy, unstressed colonies suggests a commensal relationship with the coral host. Furthermore, their presence in colonies of *P. clavata,* which have been described as heat-resistant, raises questions about their possible role in coral heat resistance. Another group of microeukaryotes which are frequently found in corals are the apicomplexan corallicolids. These alveolates are associated with 70% of the coral genera studied with a relative abundance sometimes as high as that of the Symbiodiniaceae [4, 16, 17]. Despite their heterotrophic behavior and role as human pathogens, such as in the malaria disease, corallicolids appear to be commensals in corals, as they are widely distributed and present in healthy individuals [15, 17]. While pioneering studies have begun to unravel the diversity of coral-associated microeukaryotes, further research is necessary to understand their role in the coral microbiome, how they respond to environmental conditions, and especially to the rapid changes in the marine environment due to anthropogenic disturbances.

Mediterranean octocorals are good models for studying host-microbiome relationships because they tend to have strong and stable associations with their microorganisms [18]. Previous studies have shown that bacteria from the Oceanospirillales family (i.e. *Endozoicomonas*) or from the Spirochaetaceae family constitute ˃50% of the bacterial community in gorgonians (i.e. *P. clavata*, *Eunicella sp*.; [17–19]) and in the precious red coral *Corallium rubrum* [20, 21], respectively. The coral microbiome remains highly stable through time and space, except during thermal stress when the coral health is impaired [22–27]. Research has therefore linked the health of the Mediterranean corals to the decline in the main bacterial symbionts and the presence of pathogenic or opportunistic bacteria [23–27]. However, only one study was conducted on the eukaryome of a Mediterranean octocoral [15]. This experimental study investigated the eukaryome of the Mediterranean gorgonian *P. clavata* prior to heat stress exposure, and subsequently recorded coral mortality rate. It was found that heat-resistant gorgonians had a eukaryome enriched with Dino-Group I Clade 1, while heat-susceptible gorgonians had a eukaryome enriched with apicomplexan corallicolids. This suggests that certain microeukaryotes, such as Dino-Group I, may contribute to holobiont fitness. However, as the study did not analyze the microeukaryotic communities during or after the heat stress event, it does not allow an understanding of the heat-induced changes occurring in the coral eukaryome.

In light of the pioneering findings by Bonacolta *et al*. [14], and given the increasing die-off of corals in the Mediterranean Sea due to ocean warming [28–30], it is crucial to further investigate changes in the microeukaryotic communities associated with corals under experimental heat stress and natural marine heatwave (MHW) events. Coral populations are facing repeated mass mortality events [28–30], caused by the increasing frequency, duration, and intensity of MHWs due to climate change [31–34]. In 2022, the northwestern Mediterranean Sea experienced three to seven MHWs between May and September, leading to local sea surface temperature anomalies of up to 4.3°C above the summer average and exceeding the previous maximum of +2–3°C recorded during the 2003 MHWs [35–37]. The harmful impacts of the MHWs have been mostly felt above 30 m depth, where the Mediterranean seawater temperatures exceeded 23°C–24°C and affected coral health [26]. In contrast, coral populations living below 30 m depth remained relatively unaffected by MHWs as the temperature did not exceed 23°C [37].

To better understand the impact of heat stress on the coral eukaryome, we studied the changes occurring in the microeukaryotic communities associated with the Mediterranean red coral *C. rubrum* (Linnaeus, 1758) during the 2022 MHWs. Together with other octocoral species, *C. rubrum* is one of the ecosystem engineer species of the Mediterranean marine animal forests, which provide complex habitats for numerous organisms and therefore contribute significantly to biodiversity [38–40]. We collected *C. rubrum* samples at different locations from colonies affected by the MHWs of the summer 2022 (16–28 m depth) and from mesophotic colonies deep enough to escape MHWs (35–37 m depth). We also collected control colonies in winter 2023. Using an *18S rRNA* gene metabarcoding approach with specific primers which excluded the red coral *18S rRNA* sequences, we aimed to gain a comprehensive overview of the eukaryome of *C. rubrum* and assess the impact of thermal stress disturbances on the associated microeukaryotic communities.

## Material and methods

### Study areas, sample collection, and biological material

**Figure 1:**
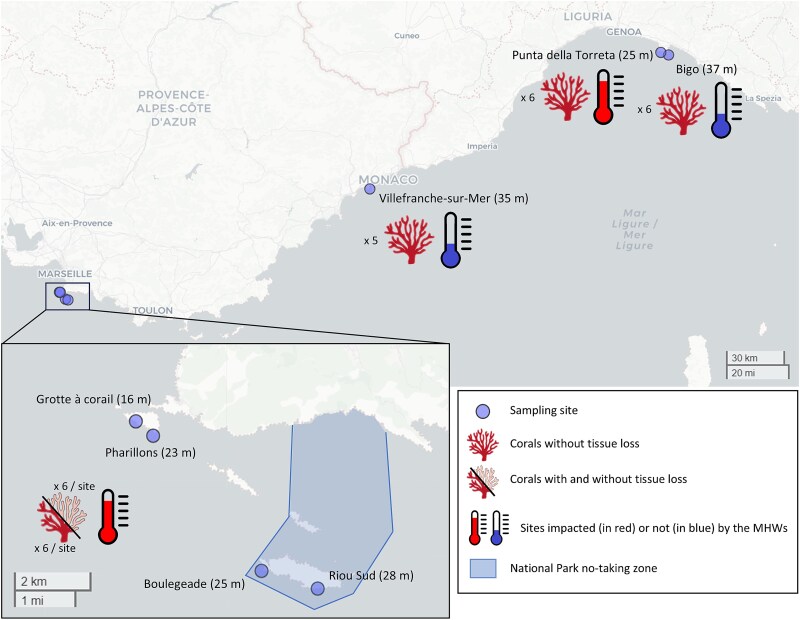
Locations of the different sampling sites. Colony health condition (with or without tissue loss) and intensity of the MHWs are indicated for each site.

**Figure 2:**
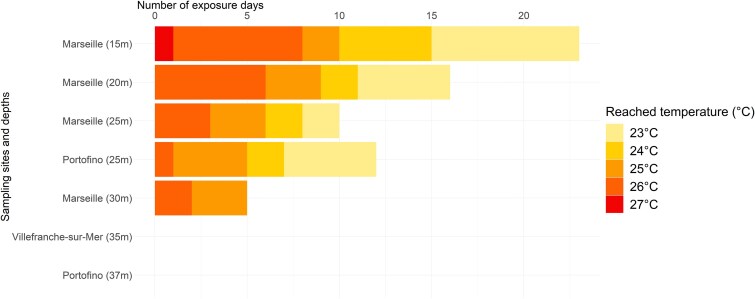
Number of days for which temperatures reached 23°C in the different sampling sites and depths, in 2022 (Marseille, Portofino) or 2023 (Villefranche-sur-Mer). Values of seawater temperature were extracted from the T-MEDNet database (www.t-mednet.org) at Riou Sud (Calanques National Park, France, 2022) and Portofino (MPA, Italy, 2022) and from Somlit (www.somlit.fr) at Villefranche-sur-Mer (point B, France, 2022 and 2023).

Sampling was conducted at the end of the summers of 2022 (September–November) and 2023 (November), after severe MHW events at three locations in the NW Mediterranean Sea, within the photic (from 16 m to 28 m depth) and mesophotic zone (from 35 to 37 m depth; Fig. 1). Corals of different health conditions were collected depending on the site (as detailed below). Hourly seawater temperature measurements in these locations were extracted from the T-MEDNet database (www.t-mednet.org) and from Somlit (www.somlit.fr), covering the entire depth range (Fig. 2). During the summer, seawater temperatures above 30 m depth frequently exceeded 23°C–24°C, which are known to be detrimental to the health of *C. rubrum* (Fig. 2; ref. [26]). In contrast, temperatures below 30 m depth remained below 23°C. Thus, mesophotic sites below 30 m depth were designated as control sites, while sites above 30 m depth were considered as MHW-impacted sites. Red coral colonies collected from the latter sites were considered heat-stressed colonies.

Heat-stressed samples of *C. rubrum* were collected in MHW-impacted sites (<30 m) in September 2022 in the Calanques National Park (Marseille, France) and in October 2022 in the *Portofino* Marine Protected Area (Italy), with the consent of the respective park authorities (Fig. 1). In Marseille, 48 individual samples of *C. rubrum* (3 cm long), showing different health statuses (0% or 90% tissue loss), were collected at four sampling sites: *Grotte à corail* (16 m depth), *Pharillons* (23 m depth), *Boulegeade* (25 m depth), and *Riou Sud* (28 m depth), according to the protocol described in Prioux *et al*. [24]. At these sites, temperatures reached 23°C during 23 days at 15 m depth and 5 days at 30 m depth (*Riou Sud*, Calanques National Park, Marseille, France; Fig. 2).

In Portofino, at the MHW-impacted site *Punta della Torreta* (25 m), six samples of 3 cm in length from different colonies were collected, which showed no tissue loss. At this location and depth, corals experienced 12 days with temperatures exceeding 23°C (Marine Protected Area, Portofino, Italy; Fig. 2). Additionally, six replicates of 1 l seawater were collected and filtered through 0.2 μm Whatman Nuclepore Track-Etched filters (Sigma-Aldrich), with the retentate stored in RNAlater at 4°C.

Two sets of control samples of *C. rubrum* were collected in the mesophotic zone (>30 m; control sites or sites unaffected by MHWs) in the *Portofino* Marine Protected Area (MPA, Italy) in October 2022 (concurrently with the samples collected at <30 m depth) and in *Villefranche-sur-Mer* (France) in November 2023 (Fig. 1). In *Portofino*, six samples of 3 cm in length from different colonies with no signs of tissue loss were collected at the site *Bigo* (37 m), where temperatures remained below 23°C in 2022 (MPA, *Portofino*, Italy; Fig. 2). Six replicates of 1 l seawater were also collected and filtered through 0.2 μm Whatman Nuclepore Track-Etched filters (Sigma-Aldrich), with the retentate stored in RNAlater at 4°C. In *Villefranche-sur-Mer,* five samples of 3 cm in length, with no signs of tissue loss, were collected at a depth of 35 m, where temperatures remained below 23°C in both 2022 and 2023 (Point B, *Villefranche-sur-Mer*, France; Fig. 2). All coral samples were immediately placed in separate Ziploc® plastic bags containing natural seawater after collection. Onboard, the samples were transferred to Eppendorf tubes, flash-frozen in liquid nitrogen (~190°C, ©CX100 Worthington Industries), and then stored at −80°C in the lab until further processing.

### DNA extraction

Genetic material was extracted from ⁓1.5 cm of tissue using the DNeasy PowerBiofilm kit (QIAGEN, Hilden, Germany) with some adjustments. During cell lysis, 2 μl of proteinase K (600 U/ml) was added to each sample, followed by an incubation period of 2 h at 60°C. Subsequently, bead beating was performed for 2 min using the CryoMill (Retch, Germany) set at a frequency of 30 Hz at room temperature. DNA concentration was determined using a Qubit fluorometer, and the extracted DNA was stored at −20°C.

### MiSeq amplicon sequencing of the *18S rRNA* gene

#### Design of primers targeting the eukaryotic V4 region and minimizing host signal

Previous studies on the coral microbiome [14, 15, 41] have shown that the different universal *18S rRNA* gene primer pairs amplify the coral host sequences over the microeukaryotic sequences, which are in fewer numbers. We tested the universal *18S rRNA* gene primers TAReuk454FWD1 (5′-CCAGCASCYGCGGTAATTCC-3′) and TAReukREV3 (5′-ACTTTCGTTCTTGATYRA-3′), which target the V4 region [42], and mainly obtained reads corresponding to the red coral sequences (99.96% of reads were coral-derived; Supplementary File S2, Supplementary Table S1 and Supplementary Fig. S1).

To overcome this bias, we used customized primers derived from the UNonMet-PCR primers targeting the V4 region of the *18S rRNA* gene, initially described in Bower *et al.* [43] and further validated for Illumina amplicon sequencing by del Campo *et al.* [41]. Originally, the UNonMet-PCR primers were designed to reduce the signal from metazoan hosts while amplifying that of microeukaryotes. Because these primers did not successfully reduce the signal from *C. rubrum* (Supplementary File S2, Supplementary Table S1), new primers, named 18S-NonMet_Cr-F (5′-AAGTCTGGTGCCAGCASCC-3′) and 18S-NonMet_Cr-R (5′-TTTAAGTTTCAGCCTTGCGAT-3′) were designed. The NonMet_Cr-F primer was fully designed, while the 18S-NonMet_Cr-R primer was modified from the UNonMet-PCR reverse primer. We used a manually curated database containing 544 *18S rRNA* gene sequences from a diverse array of eukaryotes, including *C. rubrum* and microeukaryotic lineages previously detected in coral microbiota and available from the nr (non-redundant) nucleotide database of NCBI. Each nucleotide in the designed primers was carefully screened to ensure maximum dissimilarity to *C. rubrum* sequences within the V4 region while simultaneously maintaining universality to target microeukaryote sequences, particularly paying attention to lineages yet described as being part of coral microbiomes in previous literature. The alignment of the primers on the database is available as Supplementary File S1.

*In silico* PCRs were done against the sequences from the PR2 microeukaryotic database (https://app.pr2-primers.org/pr2-primers/; v. 2.0.0) using the newly designed primers 18S-NonMet_Cr to test their universality in targeting the different microeukaryotic taxonomic groups. A sequence was considered amplified by the primers set if each primer had ≤ two mismatches with the sequence, and the percentage of sequences amplified for each microeukaryotic taxonomic group was calculated. As a comparison, the same analysis was done using the universal primers TAReuk454FWD1 and TAReukREV3 known to amplify the *18S rRNA* gene V4 region of both microeukaryotes and eukaryotes [42].

#### Library preparation and sequencing

PCR reactions were performed on coral and seawater samples along with three negative controls. They were done in a total volume of 25 μl. The reaction mixture comprised 5 μl of the 5X Q5® reaction buffer, 0.5 μl of dNTPs, 1.25 μl of each of the forward and reverse primers with Illumina adaptors (10 μm), 0.25 μl of the Q5® High-Fidelity DNA Polymerase, and 2 μl of genomic DNA (or DNA-free water for negative control). Cycle parameters involved an initial denaturation at 98°C for 30 s, followed by 35 cycles of denaturation at 98°C for 10 s, annealing at 63°C for 30 s, and extension at 72°C for 30 s, with a final elongation step at 72°C for 2 min. For comparison purposes, PCRs using the universal *18S rRNA* primers [42] with Illumina adaptors were also done on the DNA of four coral samples from the dataset. An agarose gel electrophoresis was performed on the PCR products to confirm successful amplification (Supplementary Fig. S2). DNA fragments with the expected size (~500–730 bp) were excised from the gel and purified using the GFX™ PCR DNA and Gel Band Purification kit (Illustra™). DNA concentration was determined using a Qubit fluorometer and amplicons were sent to STAB-VIDA (Portugal) for a paired-end sequencing (2 × 300 bp, 600 cycles) with the V3 chemistry on an Illumina MiSeq platform. All further steps of the amplicon library preparation were conducted using Illumina’s standard “16S Metagenomic Sequencing Library Preparation” protocol [44]. The FASTQ files containing the raw sequencing data have been deposited in the NCBI’s Short Read Archive under the BioProject accession number PRJNA1147612.

#### Bioinformatics data processing

The *18S rRNA* gene amplicon data were processed using the DADA2 pipeline (version 1.16; https://benjjneb.github.io/dada2/index.html; ref. [45]). Sequencing resulted in 7 899 126 reads ranging from 51 974 to 153 314 reads per sample (Supplementary File S2, Supplementary Table S2). Negative controls contained from 1104 to 123 664 reads per sample. Primer sequences were trimmed at the 5′-end of each read using Cutadapt v3.1 [46].

Read quality profiles were inspected, and reads were filtered and trimmed with the following settings: maxN = 0, rm.phix = TRUE, maxEE = c(2,2), truncLen = c(298,240). As the size of most of the amplicons exceeded 600 bp (Supplementary Fig. S3), only 10% of reads successfully merged on average, with 35% of the samples having a merging ratio below 5% (Supplementary File S2  Supplementary Table S3). Following this observation, it was decided neither to merge nor to use R1 reads only but to concatenate R1 and R2 reads using mergePairs(justConcatenate = TRUE) as it could offer a slight improvement in taxonomic resolution [47]. Error rates were computed and used for sequence inference. An amplicon sequence variant (ASV) table was constructed based on concatenated and de-noised reads. Chimeras were detected and removed from the table using the removeBimeraDenovo() function. The number of reads and sequences per sample passing through the different steps of the pipeline is presented in Supplementary File S2, Supplementary Table S3.

Taxonomy was assigned to the 14 495 ASVs using the assignTaxonomy() function and the PR2 reference database v4.13.0 [48]. The ratio between the number of sequences assigned to microeukaryotic ASVs (i.e. removing sequences corresponding to Metazoa ASVs) and all sequences was calculated for both primer sets (i.e. the newly designed 18S-NonMet_Cr primer set and the universal *18S rRNA* gene primer set TAReuk454FWD1 and TAReukREV3).

ASVs corresponding to Metazoa and Embryophyceae were then removed from the dataset, resulting in 8188 ASVs across eighty samples (Supplementary File S2, Supplementary Table S4). The ASV table and metadata are available in Supplementary File S2 as Supplementary Tables S5 and S6, respectively, and the sequences of the ASVs are available as Supplementary File S3. Highly abundant ASVs from coral samples (ASV4, ASV5, ASV13, ASV18, ASV20, ASV21, ASV40, ASV41, ASV49, and ASV71) underwent BLAST analysis to assess their similarity to previously documented sequences deposited in the nr nucleotide database of NCBI. Detailed results from these analyzes can be found in Supplementary File S4.

#### Analysis of the microeukaryotic 18S rRNA gene sequencing data

All statistical analyzes were conducted in the R environment (version 4.2.1) using the R-package *phyloseq* [49]. The R-package *decontam* [50] was used to identify contaminant ASVs in the samples based on the negative control samples (isContaminant function); however, no ASVs were identified as contaminants. Observed species richness and evenness (Shannon index) were calculated using the R-package *vegan* [51].

Alpha diversity analyzes were conducted on the unrarefied ASV count table, given that rarefaction curves mostly reached the plateau (Supplementary Fig. S4) and the current debate concerning the applicability of unrarefied versus rarefied data [52, 53]. Differences in observed richness and Shannon index were explored by fitting a generalized linear model using a negative binomial distribution and a normal distribution, respectively. Two models were considered: one including the sampling site as a fixed factor on samples without tissue loss and another including both sampling sites and tissue loss conditions as fixed factors for samples collected in Marseille. The interaction term was excluded from consideration due to its lack of significance (Supplementary File S2, Supplementary Table S7). *Post hoc* tests were performed to identify potential pairwise differences (R-package *emmeans*, [53]).

To examine potential changes in the composition of the microeukaryotic communities of *C. rubrum* across sampling sites and tissue loss conditions, compositional data-based analyzes [54–57] were used. The raw ASV abundances underwent a centered log-ratios (clr) transformation using the R-package *compositions* [58]. Zero counts were imputed via Bayesian multiplicative replacement (Bayes-LaPlace BM method) using the cmultRepl() function in the R-package *zCompositions* [59]. An Aitchison distance matrix was constructed by computing the Euclidean distance between samples using the clr-transformed data table. Principal component analyzes and permutational multivariate analysis of variance (performed with the adonis() function from the R-package *vegan*) were carried out to assess variations in the microeukaryote community structure between groups of sites, i.e. control versus MHW-impacted sites and between deep (≥25 m depth) versus shallow (<25 m depth) MHW-impacted sites, and between colonies without and with 90% tissue loss (Supplementary File S2, Supplementary Table S8). Subsequently, potential pairwise differences were examined using the permanova_pairwise() function from the R-package *ecole* [60], with *P*-values adjusted for false discovery rate (Supplementary File S2, Supplementary Table S8).

All graphs for alpha and beta diversity were done using the R-package *ggplot2* [61]. For the representations of the composition of the communities (i.e. barplot and bubble plot), replicates of each Condition-Site couple were merged by averaging the counts of each ASV or family across all samples using the merge_samples() function from the R-package *phyloseq* [49]. Following this, the transform_sample_counts() function was applied to normalize the data, converting raw counts into relative abundances.

Differential abundance analyzes were performed to identify ASVs that were differentially abundant between sampling sites and tissue loss conditions using the R-package *ANCOM-BC* (59; version 02–2023; ancombc2() function using prv_cut = 0.1 and alpha = 0.05; pairwise = TRUE for sampling sites; Supplementary File S2  Supplementary Tables S9 and S10). The core eukaryome of the red coral colonies showing no tissue loss was identified using the core_members() function at the ASV level (arguments: detection = 0.01, prevalence = 98/100, include.lowest = FALSE) from the R-package *microbiome* [62] and a custom-made function with the R-package *dplyr* [63] for other taxonomic levels (Supplementary File S2, Supplementary Table S11).

To provide a phylogenetic context for the eight most prevalent taxonomic families (i.e. Dino-Group I Clade 1, Licnophoridae, Warnowiaceae, Corallicolidae, Exobasidiomycetes, Ephelotidae, Labyrinthulaceae, and Philasterida), we did a multiple sequence alignment (MSA) using the Super5 algorithm in Muscle5 [64] for each family (Supplementary File S2, Supplementary Table S12). The MSAs included the most abundant ASVs of each family (those representing >50% of the total microeukaryotic community in at least one coral sample), public ASV sequences obtained from a BLAST analysis (98% similarity threshold) of the representative ASV sequence of each family (i.e. the most abundant ASV for each family) on the nr nucleotide database of NCBI, and, when available, ASV sequences documented in other coral studies and annotated as belonging to the same microeukaryotic families [4, 14]. All trees were rooted with an ASV sequence (or a sequence from NCBI when it was not available) of an outgroup selected as belonging to the same taxonomic order but from a different family (Supplementary File S2, Supplementary Table S12). PhyML [65] was used to calculate the phylogenetic distances between the ASV sequences and construct rooted trees based on the maximum-likelihood principle. Different substitution models were selected (Supplementary File S2, Supplementary Table S12) according to the microeukaryotic family using the Smart Model Selection software implemented in the PhyML environment [66] with the Akaike’s Information Criteria. To ensure statistical consistency, the non-parametric variant of approximate likelihood-ratio test (SH-aLRT; [63]) was applied to the datasets. The resulting trees were modified for publication using ggtree [67]. In addition, the percentage of similarity between the representative ASVs from each family and the ASV sequences documented in the other coral studies and belonging to the same microeukaryotic families [14, 15, 68] was calculated using the NCBI blastn suite-2sequences website.

## Results

### Specificity and universality tests on the newly designed primers

The non-metazoan primers designed specifically for *C. rubrum* notably reduced the coral host signal compared to the universal eukaryotic *18S rRNA* gene primers ([43]; Supplementary Fig. S1). Indeed, they yielded an average ratio of 22% microeukaryotic sequences per sample, markedly higher than the 0.04% ratio obtained with universal primers (Supplementary File S2; Supplementary Table S1; Supplementary Fig. S1). Rarefaction curves indicated that these primers were efficient in characterizing the microeukaryotic communities associated with *C. rubrum*, as most curves reached the plateau (Supplementary Fig. S4). Results of the *in silico* PCRs on the PR2 database of microeukaryotes indicated that the 18S-NonMet_Cr primers successfully amplified ˃84% of the sequences of most microeukaryotic groups when setting the maximum number of mismatches per primer to two (>60% of sequences amplified for all taxonomic groups; Supplementary Fig. S3). However, our primers only amplified 40%, 45%, and 60% of the sequences corresponding to the microeukaryotic species from the Obazoa and Excavata supergroups, as well as from the Eukaryota_X group (i.e. unclassified microeukaryotes), respectively. Therefore, our primers might be less universal for these groups than the universal *18S rRNA* gene primers (V4 region; Supplementary Fig. S3). After sequencing, using the 18S-NonMet_Cr primers, we captured a wide range of microeukaryotic groups, resulting in 8187 different ASVs spanning across 4 domains, 30 divisions, 131 classes, 261 orders, and 479 families of microeukaryotes (Supplementary File S2; Supplementary Table S4). It included groups that have already been found associated with other coral species, such as members of the Alveolata division, including dinoflagellates, ciliates, and apicomplexans.

### Overview of the eukaryome of *C. rubrum*

Microeukaryotic communities were structurally different between the coral and seawater samples, although both communities were predominantly dominated by the Alveolata division (F = 2.7; d.f. = 1; *P* = .016; Supplementary Fig. S5 and Supplementary File S2; Supplementary Table S7). The microeukaryotic communities associated with corals contained on average 10 times less ASVs than seawater (94 ASVs and 1157 ASVs per sample on average in the coral and seawater samples, respectively; Supplementary File S2; Supplementary Table S6).

**Figure 3:**
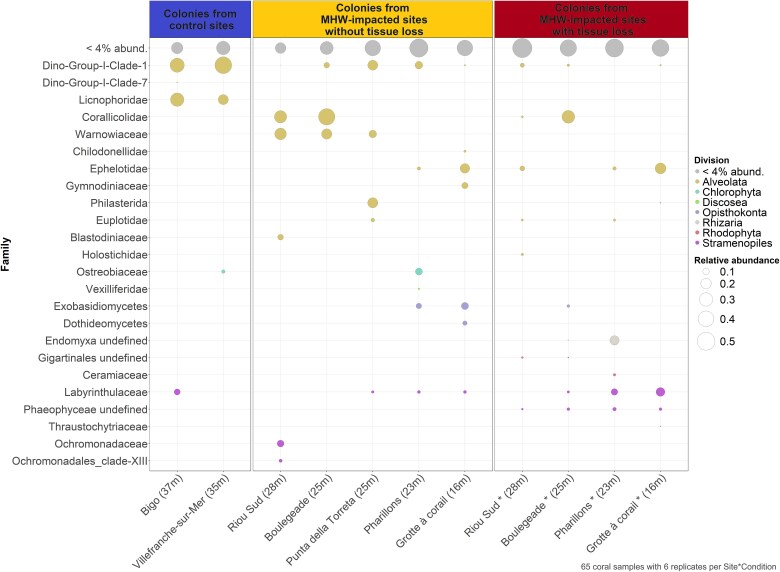
Relative abundance of the microeukaryotic families associated with *C. rubrum* colonies depending on the colony health condition (0% or 90% tissue loss) and collection site. Microeukaryotic taxa are ordered by taxonomic division for those with an average relative abundance above 1%.

**Figure 4:**
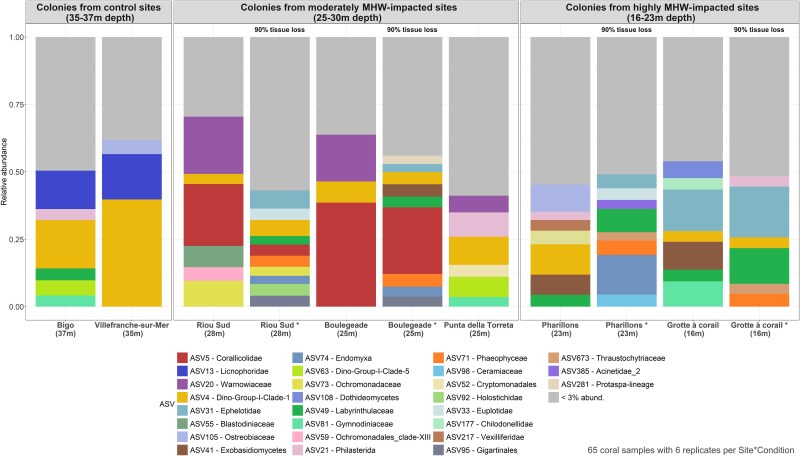
Composition of the microeukaryote communities associated with *C. rubrum* colonies (ASV level). Relative abundance and taxonomy of the most abundant microeukaryote ASVs associated with *C. rubrum* colonies of different health conditions and collected at different sites. Microeukaryotic taxa with an average relative abundance below 3% are aggregated.

The eukaryome of *C. rubrum* was not significantly different between the control sites (*Bigo* and *Villefranche-sur-Mer*, pairwise analysis; F = 0.4; *P* = .8; Supplementary File S2; Supplementary Table S11) and was composed of microeukaryotes from the Dino-Group I and Licnophoridae, which collectively formed ~45.5% of the total community (Figs. 3 and 4). Overall, colonies without tissue loss were consistently associated with Dino-Group I, though at lower abundances than in control sites, as well as with Gymnodiniales, Labyrinthulomycetes (Stramenopiles division), Corallicolidae, Ephelotidae, and Exobasidiomycetes, with the association of the latter three varying depending on the sampling sites. In colonies exhibiting tissue loss, the relative abundance of Labyrinthulomycetes increased significantly, reaching up to 12% in some sites (Figs. 3 and 4). Additionally, there was an emergence of Phaeophyceae, averaging 5% in these colonies (Figs. 3 and 4).

### Changes in the microeukaryotic communities associated with heat-stressed colonies without tissue loss

**Figure 5:**
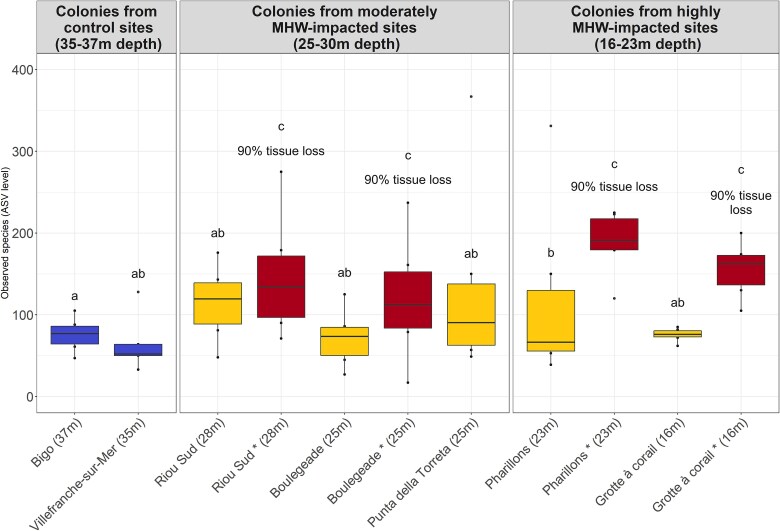
Microeukaryote species richness associated with *C. rubrum* colonies depending on the collection site and the colony health condition. Colonies collected at the control sites are represented in blue. Heat-stressed colonies without tissue loss but collected in MHWs-impacted sites are represented in yellow, while those with tissue loss are represented in red. Significant statistical differences (*P* < .05) between groups are indicated by letters.

**Figure 6:**
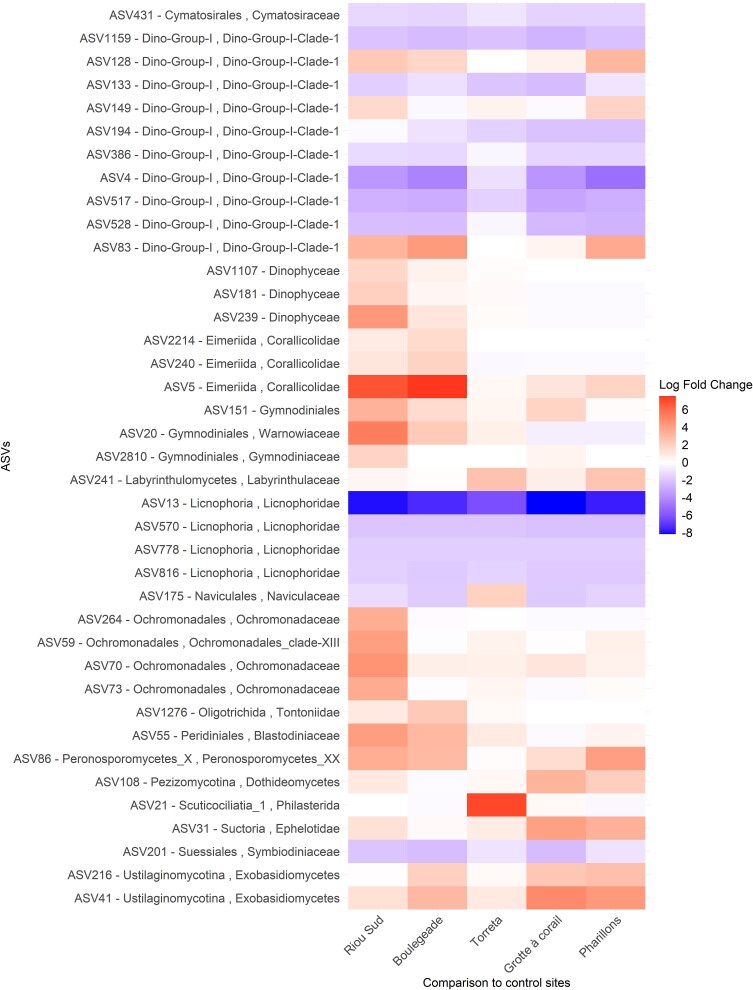
Microeukaryote ASVs differentially abundant (ANCOM-BC results) between the *C. rubrum* colonies collected at the control sites (*Villefranche-sur-Mer* and *Bigo*) and the *C. rubrum* colonies collected at the other sampling sites. Differences in relative abundance calculated as log fold change (natural log).

While the observed species richness and Shannon index were not significantly different between colonies collected at the control and MHW-impacted sites (Fig. 5), the structure of the microeukaryotic communities differed (F = 3.8; d.f. = 1; *P* = .001; Figs. 3, 4, and S6, Supplementary File S2; Supplementary Table S7 and S8). Differential abundance analyszs identified 39 ASVs whose relative abundance was different between the colonies collected in the control and MHW-impacted sites (Fig. 6). ASV4 and ASV517-Dino-Group I Clade 1*,* as well as ASV13-Licnophoridae, exhibited significantly lower relative abundances in colonies sampled in MHW-impacted sites compared to the colonies at control sites. ASV4 and ASV517-Dino-Group I Clade 1 relative abundances collectively decreased from 35% to 3%, and the ASV13 relative abundance decreased from 22% to 1% on average from control to heat-stressed colonies (Fig. 6, Supplementary File S2; Supplementary Tables S5 and S9). On the contrary, other ASVs were significantly more abundant in colonies collected in the MHW-impacted sites, such as ASV41-Exobasidiomycetes and ASV86-Peronosporomycetes, whose relative abundances increased from 0.006% and 0.02% on average in the control colonies to 3% and 0.9% on average in the heat-stressed colonies, respectively (Fig. 6 and Supplementary File S2; Supplementary Tables S5 and S9).

Different microeukaryotes dominated the communities depending on the location and depth. Colonies from the deepest sites (≥25 m; *Riou Sud*, *Boulegeade* and *Punta della Torreta*) were characterized by a higher proportion of Warnowiaceae and especially ASV20 (18% on average in the deep MHW-impacted sites versus and 2% in control sites*;*  Figs. 4, 6, and7). Furthermore, ASV21-Philasterida was in a higher proportion in colonies from *Punta della Torreta* (20% on average), while ASV5-Corallicolidae was relatively abundant in colonies collected in *Riou Sud* (20% on average) and *Boulegeade* (60% on average; Figs. 4, 6, and7). In the shallowest sites (<25 m; *Pharillons* and *Grotte à corail*), the microeukaryotic communities showed a higher relative abundance of ASV41-Exobasidiomycete (*Malassezia* genus) and ASV31-Ephelotidae (Figs. 4, 6, and7).

**Figure 7:**
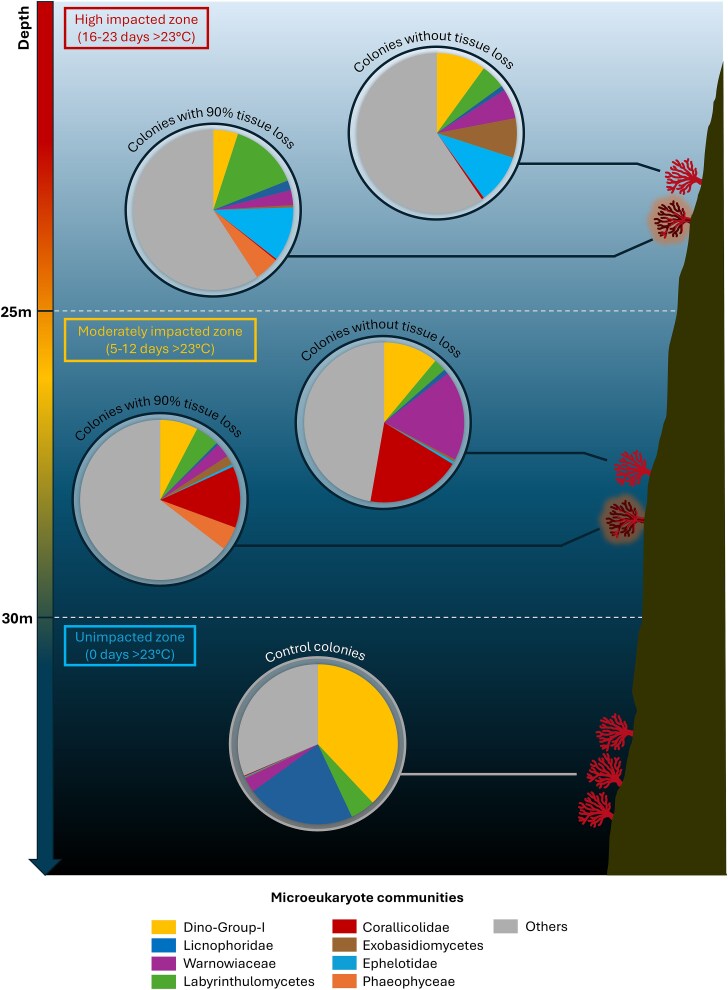
Schematic representation of the composition of the microeukaryote communities associated with *C. rubrum* colonies according to the sampling site and coral health condition. Only the most prevalent microeukaryote groups are represented.

### Changes in the microeukaryotic communities associated with colonies showing tissue loss

**Figure 8:**
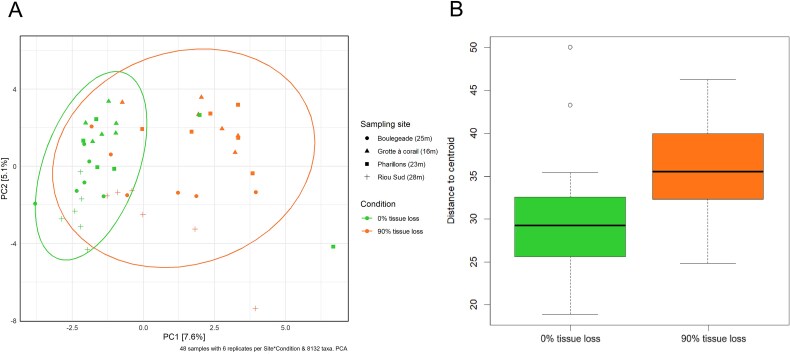
Structure of the microeukaryote communities associated with *C. rubrum* colonies with 0% and 90% tissue loss in the Calanques National Park (Marseille). (A) Principal component analysis of the Aitchison distance matrix based on the composition of the microeukaryote communities (ASV level), showing the distribution and dispersion of the *C. rubrum* samples collected at the different sites. (B) Beta-dispersion assessment using the distance to centroids of samples with and without tissue loss.

At the MHW-impacted sites of the Calanques National Park in Marseille, where most colonies with 90% tissue loss were collected, a higher species richness in microeukaryotes was observed in the colonies with tissue loss compared to the ones without tissue loss (1.6 times more on average; estimate std. = 0.49; error = 0.13; *z*-value = 3.6; *P* = .0003; Fig. 5 and Supplementary File S2; Supplementary Table S7). Furthermore, the composition of the communities between the colonies with and without tissue loss differed (F = 1.75; d.f. = 1; *P* = .001; Fig. 8 and Supplementary File S2; Supplementary Table S8), with a higher beta-dispersion of the samples corresponding to the colonies with tissue loss (F = 12.2; d.f. = 1; *P* = .001; Fig. 8 and Supplementary File S2; Supplementary Table S8). The differential analysis identified eight differentially abundant ASVs in the colonies with tissue loss, including three Phaeophyceae ASVs (ASV71, ASV184, and ASV394) and three Labyrinthulaceae ASVs (ASV252, ASV389, and ASV691) whose relative abundance increased (Supplementary File S2; Supplementary Table S10). Analyzes on each site showed that a difference in composition between colonies with and without tissue loss was observed in *Riou Sud* and *Boulegeade*, but not in *Pharillons* and *Grotte à corail* (Supplementary File S2; Supplementary Tables S8 and S10).

### Phylogenetic analyzes of *C. rubrum* associated microeukaryotes

Phylogenetic analyzes were done on the ASVs belonging to microeukaryotic taxa found in high relative abundances in control colonies (i.e. Dino-Group I Clade I and Licnophoridae), as well as in heat-stressed colonies (i.e. Warnowiaceae, Corallicolidae, *Malassezia*, Ephelotidae, and Labyrinthulaceae).

ASV4 from the Dino-Group I Clade 1 clustered together and with Dino-Group I sequences recovered from the Mediterranean octocoral *P. clavata* (98.42% sequence similarity) and the tropical hexacoral *P. damicornis* (100% sequence similarity; Supplementary Fig. S7, Supplementary File S2, Supplementary Table S12 and Supplementary File S4). ASV13-Licnophoridae clustered with the other ASVs recovered from red coral as well as from *P. clavata* and was assigned as Licnophoridae (Supplementary Fig. S8, Supplementary File S2, Supplementary Table S12, and Supplementary File S4). ASVs assigned to the Warnowiaceae family (ASV18 and ASV20) clustered with dinoflagellate sequences and especially to sequences of seawater *Warnowia* spp. (99%–100% sequence similarity; Supplementary Fig. S9, Supplementary File S2, Supplementary Table S12 and Supplementary File S4). ASV5-Corallicolidae sequence was 100% similar to an unclassified microeukaryotic sequence recovered from the tropical hexacoral *Porites astreoides* and 99.21% similar to the sequence of a Corallicolidae ASV found in association with the Mediterranean octocoral *P. clavata* (14; Supplementary Fig. S10, Supplementary File S2, Supplementary Table S12 and Supplementary File S4). ASV41-*Malassezia* sequence showed 100% similarity to *Malassezia* spp. sequences described as pathogens of the skin of humans and terrestrial animals (NCBI nr nucleotide database, 66). Exobasidiomycetes (*Malassezia* genus) and Ephelotidae ASVs respectively clustered with sequences of *Malassezia* spp. (98% sequence similarity) and *Ephelota* spp. (95% sequence similarity), both associated with the Mediterranean octocoral *P. clavata* (Supplementary Figs. S11 and S12; Supplementary File S2, Supplementary Table S12 and Supplementary File S4). ASV49-Labyrinthulaceae sequence was 98% similar to a *Labyrinthula* spp. sequence recovered from seagrass lesioned tissue (Supplementary File S2, Supplementary Table S12 and Supplementary File S4; 67). The closest public sequences to the other Labyrinthulaceae ASVs were Stramenopiles sequences recovered from the hexacoral *Favia* spp. (95% sequence similarity; Supplementary Fig. S13, Supplementary File S2, and Supplementary Table S12 and Supp. File S4). ASV21- Philasterida was 99% similar to sequences of *Philaster* sp. recovered from diseased aquarium corals showing signs of white syndrome and rapid tissue necrosis (Supplementary Fig. S14, Supplementary File S2, Supplementary Table S12, and Supplementary File S4).

## Discussion

Using an *18S rRNA* gene metabarcoding approach, we observed that the microeukaryotic community associated with the octocoral *C. rubrum* was composed of dinoflagellates Dino-Group I and Warnowiaceae, Stramenopiles belonging to Labyrinthulomycetes, and ciliates Licnophoridae. These microeukaryotic groups were already found in other coral species. The eukaryome of *C. rubrum* was highly responsive to thermal stress, and some microeukaryotes might be bioindicators of coral stress. Our work demonstrates the importance of modifying the initial *18S rRNA*-based UnNonMet primer set designed to study the biodiversity of microeukaryotes associated with metazoan hosts to adapt them to the studied coral species and efficiently reduce the host’s signal. Here, the primers designed for *C. rubrum* significantly reduced host signal while amplifying most microeukaryotic sequences. Overall, these findings underscore the importance of monitoring microeukaryotic communities to understand coral responses to environmental stress and highlight the technical need for species-specific primer optimization.

### The eukaryome of *C. rubrum* colonies showing no tissue loss

The microeukaryotic community associated with *C. rubrum* had a lower richness and different composition than seawater, suggesting that only certain microeukaryotes associate with *C. rubrum*. Microeukaryotes found in all colonies without tissue loss were composed of dinoflagellates Dino-Group I and Warnowiaceae, ciliates Licnophoridae, and Stramenopiles belonging to Labyrinthulomycetes. These dominant groups may be essential for the functioning of *C. rubrum*, and more generally for coral holobionts. Microeukaryotes from the Dinoflagellata and Ciliophora subdivisions e.g. have been observed in association with other coral species from both temperate and tropical environments [3, 11, 14, 15]. Specifically, several Dino-Group I ASVs observed in the red coral eukaryome were closely related to Dino-Group I ASVs found in the temperate octocoral *P. clavata* [15] and the tropical hexacoral *P. damicornis* [14]. Even if the role of Dino-Group I within the coral holobiont is still unclear, the Syndiniales class, to which these dinoflagellates belong, is exclusively found in marine environments and has been described as potentially parasitic due to its broad distribution across ocean food webs [69]. Warnowiaceae, other dinoflagellates from the Gymnodiniales order found in the red coral eukaryome, have been observed on sick tropical hexacorals, and were suspected to be responsible, with bacterial infection, for the black band disease in producing paralytic shellfish toxins [70–72]. Labyrinthulomycetes, a group of ubiquitous and diverse unicellular Stramenopiles, were also observed in the tissue of tropical hexacorals, but also in other marine organisms such as clams, nudibranchs, and seagrasses [68,73–80]. They are known to be involved in saprotrophic nutrient uptake [81–85]. Their osmotrophic (uptake of dissolved organic matter) and phagotrophic (feeding on algae, bacteria, and small organic matter) abilities make them ecologically important in nutrient cycling by contributing to various decomposition processes in marine ecosystems [81–85]. Some species in this family are also known to produce high levels of omega-3 polyunsaturated fatty acids, which has made Labyrinthulomycetes commercially valuable [86,87]. Further studies are needed to determine whether the Labyrinthulomycetes species that form the eukaryome of *C. rubrum* may be a nutritional symbiont that transfers lipids to the coral host. Finally, Licnophoridae ciliates have been previously observed associated with the Mediterranean octocoral *P. clavata* [15] as well as the tropical hexacoral *P. damicornis* [14]. Some Licnophoridae have been described as predators of other ciliates [88]. This relationship with other ciliates might explain their variability in relative abundance in our samples depending on the site [14].

Thus, some microeukaryotes (i.e. Dino-Group I, Warnowiaceae, and Labyrinthulomycetes) might be universal and found in association with many coral species, like the bacteria *Endozoicomonas* in the bacterial microbiome of corals [89–91]. These microeukaryotes might be relevant for the functioning of the coral holobiont and for understanding coral’s response to environmental change. Further studies investigating the eukaryome of other coral species (including temperate, tropical, and scleractinian but also octocoral species) are needed to identify universal and potentially relevant microeukaryotes in order to gain a deeper understanding of the coral holobiont.

### Microeukaryotic indicators of heat stress conditions

While species richness did not differ between the control and heat-stressed colonies, the composition of the eukaryome did (Fig. 8). Specifically, the relative abundance of Dino-Group I dinoflagellates and Licnophoridae ciliates was lower in colonies collected in the MHW-impacted sites. Dinoflagellates are sensitive to temperature fluctuations, and in scleractinian corals, e.g. the decrease in the abundance of Symbiodiniaceae dinoflagellates often marks the beginning of a deterioration in coral health [92,93]. A decline in Symbiodiniaceae abundance (commonly referred to as “coral bleaching”) is indeed systematically observed during heat stress, and corals may die if bleaching persists for a prolonged period [92,93]. The decrease in the relative abundance of the Licnophoridae ciliates in the heat-stressed corals could also be due to temperature fluctuations, as marine ciliates are generally heat sensitive as well [94,95]. Temperature increases can indirectly affect their food sources, such as bacteria and other protists, or directly affect their metabolic rate, reproduction, and survival [94,95].

In the MHW-impacted sites, the composition of the eukaryome was different depending on the sampling depth, probably because colonies were differentially affected by the thermal stress. Warnowiaceae and Corallicolidae became dominant groups in the moderately (deep) MHW-impacted sites, while Ephelotidae and the fungi Exobasidiomycetes became dominant in the highly (shallow) MHW-impacted sites (Fig. 8). While the latter, especially *Malassezia*, are known as pathogens of the skin of humans and terrestrial animals [96], it is unlikely that this change in microeukaryotic community composition is related to human proximity, and potential trophic drivers behind this change are currently unknown as for the other microeukaryotes. To identify their roles and therefore the consequences of their increase under heat stress in the holobiont functioning, these microeukaryotes need to be better characterized through other techniques such as metagenomics and cultures.

These specific microeukaryotes whose relative abundance increased in the heat-stressed colonies might be markers of the stress level that the holobiont is experiencing. They might serve as valuable bioindicators of coral health, such as certain associated bacteria [97–99], although the latter has been shown to be more reliable proxies of host health than microeukaryotes in other marine holobionts [100].

### Increased microeukaryotic diversity and epibiont proliferation in colonies with tissue loss

Colonies with tissue loss showed greater richness and beta-dispersion, alongside marked differences in the eukaryome composition, compared to colonies without tissue loss. This suggests that numerous opportunistic microeukaryotes might have taken advantage of the high level of stress of their host to settle in and potentially jeopardize coral health. This is consistent with findings on the bacterial communities of heat-stressed corals, in which opportunistic bacteria, whether saprophytic or commensal, proliferate, increasing the observed richness [101–103]. The transition to a more diverse bacterial community is a well-documented phenomenon in heat-stressed organisms, including octocorals [25,102,104,105] and specifically *C. rubrum* [24, 26]. A similar trend can be observed in the associated microeukaryotic communities under heat stress, presumably due to changes in microeukaryotic population dynamics and/or reduced control of the coral host over its microbiota. The analysis on each site showed that the compositional difference between colonies with and without tissue loss was significant in *Riou Sud* (28 m) and *Boulegeade* (25 m), but not in *Pharillons* (23 m) and *Grotte à corail* (16 m). In these shallow colonies, which do not show tissue loss yet but still highly suffer from heat, might have a eukaryome composition starting to look like the one associated with colonies with tissue loss.

Within the colonies that exhibited tissue loss, we observed the presence of microeukaryotes previously associated with diseased organisms. Labyrinthulomycetes, which are found more abundantly in the colonies with tissue loss, represent a ubiquitous and diverse group of unicellular Stramenopiles, known for their parasitic tendencies in various marine organisms [76,81,82]. They were discovered for their possible role in multifocal purple spot disease, which affects Caribbean sea fans (octocorals), although they have been found in both healthy and diseased tissues [106,107]. They probably act as opportunistic pathogens [106], engaging in “left-over scavenging” behavior following bacterial growth [81]. The sequence of the Labyrinthulomycetes ASV49 was similar to sequences of Labyrinthulomycetes found at the interface of seagrass lesioned tissue, where they grow on dead bacterial cells [108]. This behavior could be the reason for their relatively high abundance in colonies with tissue loss, where they may take advantage of the heat-induced proliferation of opportunistic bacteria [24]. Colonies with tissue loss were also associated with a higher relative abundance of Phaeophyceae macroalgae, which have been described to harbor and promote the growth of pathogenic bacteria in bleached tropical corals [109, 110]. Species of Phaeophyceae were also found as epibionts covering the necrotic branches of MHW-affected *P. clavata* colonies [111]. Considering that *P. clavata* and *C. rubrum* inhabit similar environments, it is not surprising that *C. rubrum* colonies suffering from MHWs experience the same algal proliferation.

### Suggested interactions occurring within the eukaryome of *C. rubrum*

**Figure 9:**
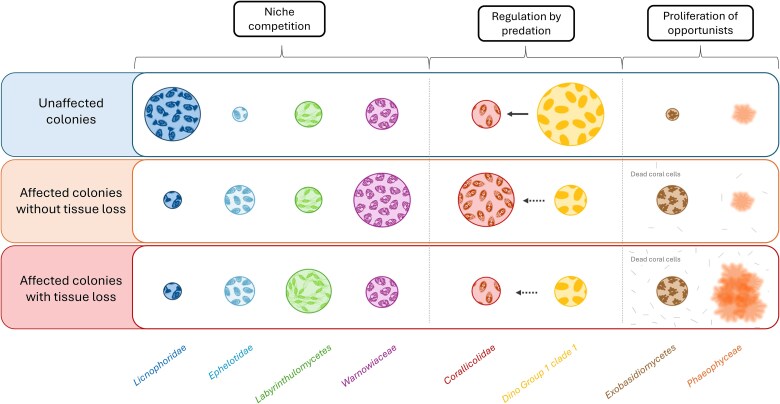
Conceptual diagram illustrating potential interrelationships and feeding dynamics among the most prevalent microeukaryote groups associated with *C. rubrum* colonies.

Microorganisms associated with *C. rubrum* are likely closely interacting through niche competition relationships. Based on the relative abundance of the dominant microeukaryotes in association with the red coral and their trophic mode inferred from their taxonomy, Fig. 9 illustrates three types (competition, predator–prey, and parasitism relations) of putative interactions that may occur between them. Licnophoridae are dominant microeukaryotes under control conditions. When the temperature increases, as these microeukaryotes are likely thermosensitive (see discussion above; [93,94]), their abundance would decrease, giving way to the proliferation of other microeukaryotes that compete for the same resources (mainly bacteria) and niche, such as Ephelotidae [6], Labyrinthulomycetes [73], and the mixotrophic Warnowiaceae [10]; (Fig. 9). In the same way, the decrease in abundance of the Dino-Group I Clade I under hot temperatures might promote the proliferation of Corallicolidae in the heat-stressed colonies. This antagonistic relationship has already been suggested within the holobiont of the Mediterranean octocoral *P. clavata* [15]. Finally, the fungi Exobasidiomycetes and macroalgae Phaeophyceae could take advantage of the compromised coral health in colonies with tissue loss to proliferate [96,111]; (Fig. 9). The assumptions above need to be validated by further studies and may be used as new research paths in our understanding of the interactions occurring between the microorganisms of the coral holobiont.

## Conclusion

The study is the first assessing the composition of the eukaryome of the Mediterranean emblematic coral species *C. rubrum*. Using an *18S rRNA* gene metabarcoding approach and newly designed primers to reduce host signal, we investigated the microeukaryotic communities associated with red coral colonies collected in unimpacted (control) and MHW-impacted sites. The eukaryome of the colonies collected at the control sites was dominated by Dino-Group I, Licnophoridae, and Labyrinthulomycetes, suggesting the physiological importance of these microeukaryotic taxa for the coral holobiont. We showed that the eukaryome composition was different between the unimpacted and MHW-impacted sites, but also between the MHW-impacted sites depending on the depth. We thus identified putative bioindicators of the heat stress level experienced by *C. rubrum* colonies during the MHW episodes. These findings enhance our understanding of the response of *C. rubrum* to the Mediterranean MHWs and the role of the microbiome in the health status of corals.

## Supplementary Material

Supp_Figures_ycaf035

Supp_file_1_Sequence_alignment_ycaf035

Supp_file_2_Tables_ycaf035

Supp_file_3_ASVs_sequences_ycaf035

Supp_file_4_NCBI_comparisons_ycaf035

## Data Availability

The datasets generated and analyzed during the current study are available in the NCBI’s Short Read Archive (SRA) under the BioProject accession number PRJNA1147612. Measurements of seawater temperature have been extracted from the public T-MEDNet database (www.t-mednet.org) and from SOMLIT (www.somlit.fr).
