# Supplementary material for: Unraveling the impact of marine heatwaves on the Eukaryome of the emblematic Mediterranean red coral Corallium rubrum
Source: ISME Commun. 2025 Feb 21;5(1):ycaf035. doi: 10.1093/ismeco/ycaf035 (PMC11894933; doi:10.1093/ismeco/ycaf035)
Supplement: Supp_Figures_ycaf035 [file supp_figures_ycaf035.pdf]

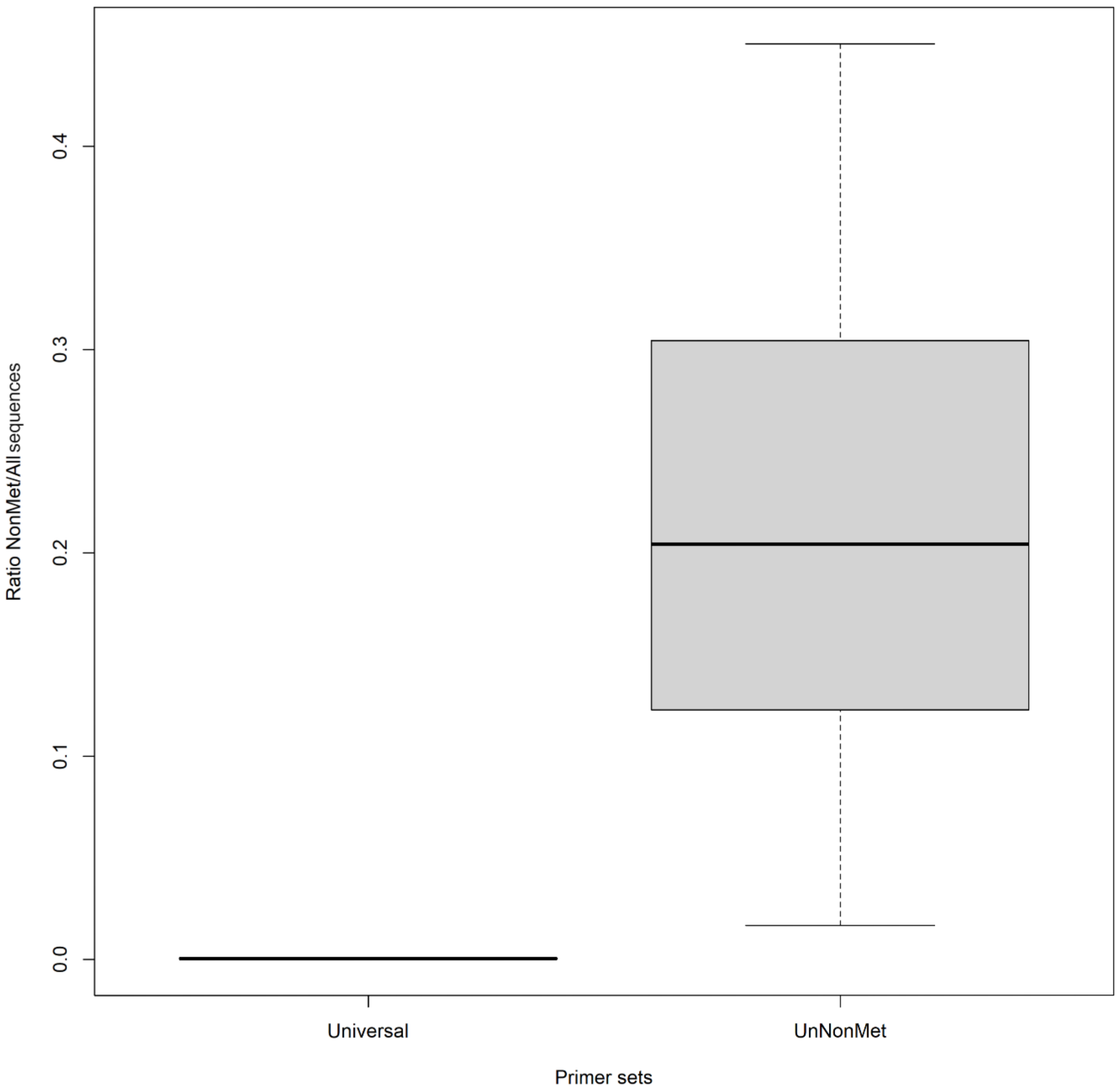

Figure S1: Ratios between the number of sequences assigned as non-metazoan and the total number of sequences (*i.e.*, non-metazoan and metazoan assigned sequences) obtained on the *C. rubrum* samples using the UnNonMet primers designed for this study and the universal *rRNA 18S* V4 primers TAReuk454FWD1 (5′-CCAGCASCYGCGGTAATTCC-3′) and TAReukREV3 (5′-ACTTTCGTTCTTGATYRA-3′), respectively. Mean of 0.04% for the universal primers (4 samples) and 22% for the newly designed UnNonMet primers (65 samples).

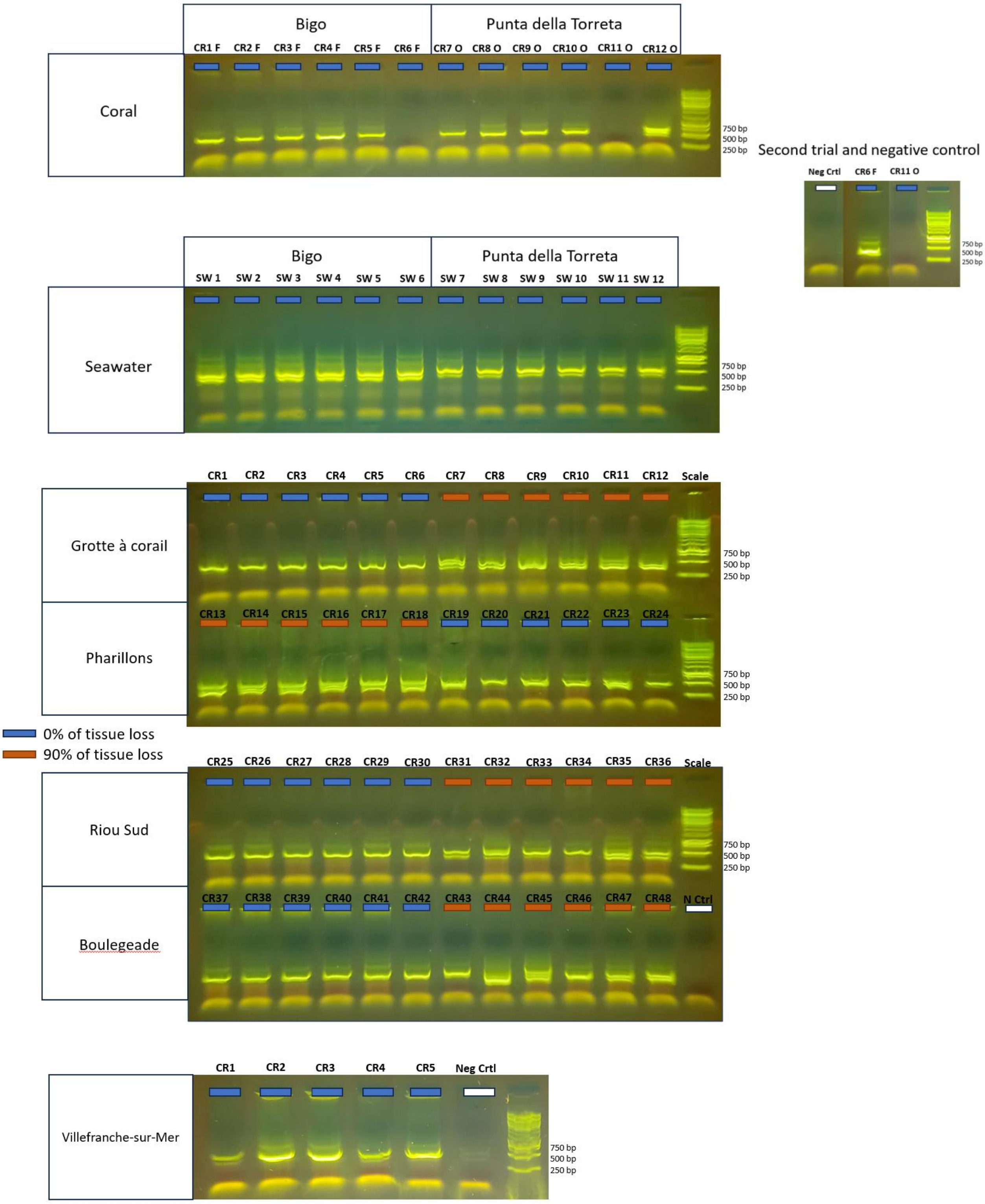

Figure S2: Images of the electrophoresis agarose gels showing the PCR products using the newly designed primers (product size between 500 and 730 bp), for each sample of the different sampling sites.

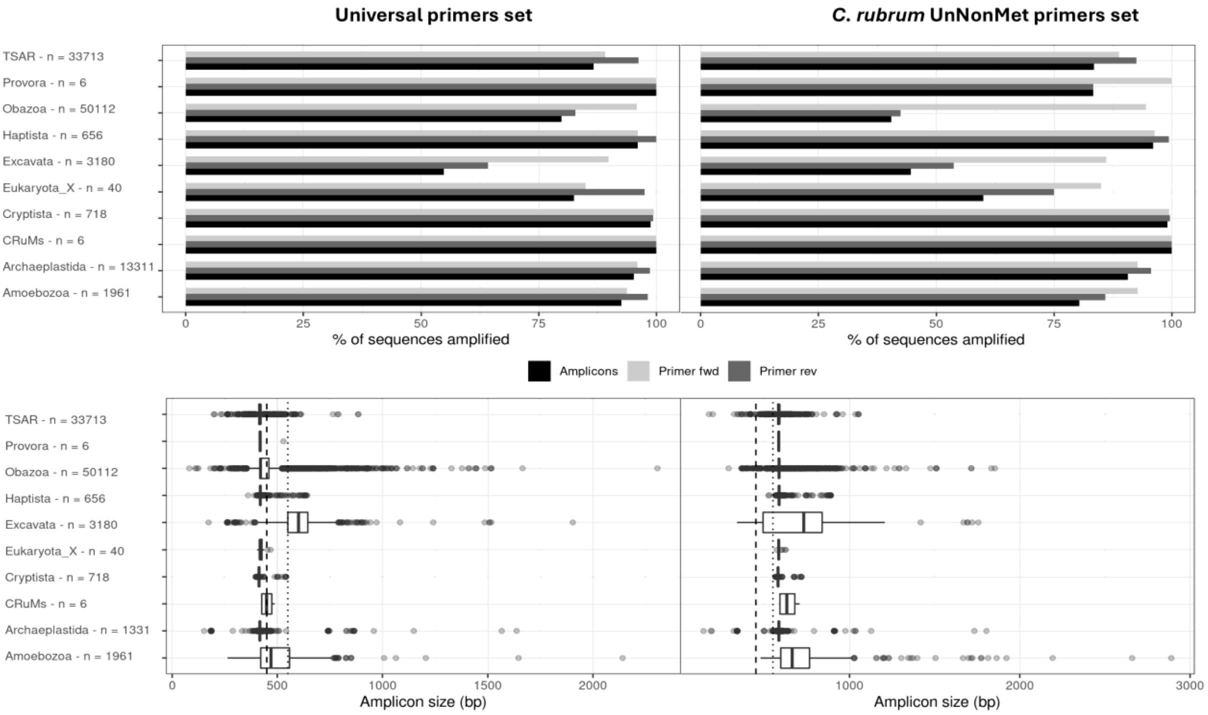

Figure S3: In-silico PCR searches. Percentage of sequences and amplicon expected size per phylum recovered from the PR2 primer database (https://app.pr2-primers.org/pr2-primers/; v. 2.0.0) using the universal *rRNA 18S* V4 primers TAReuk454FWD1 (5′-CCAGCASCYGCGGTAATTCC-3′) and TAReukREV3 (5′-ACTTTCGTTCTTGATYRA-3′) and the UnNonMet primers designed for this study. Primers were allowed a maximum of 2 mismatches.

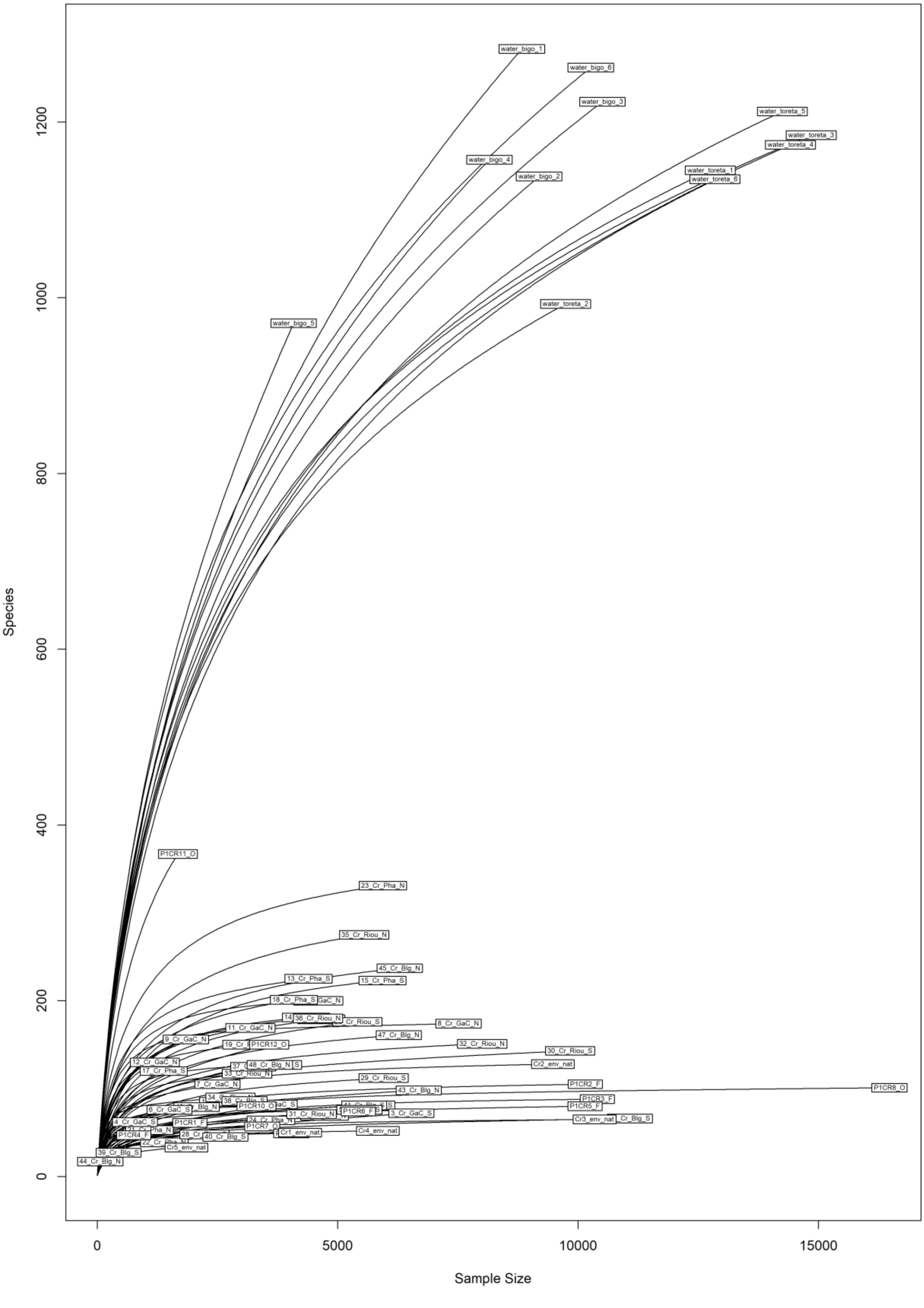

Figure S4: Rarefaction curves for all samples.

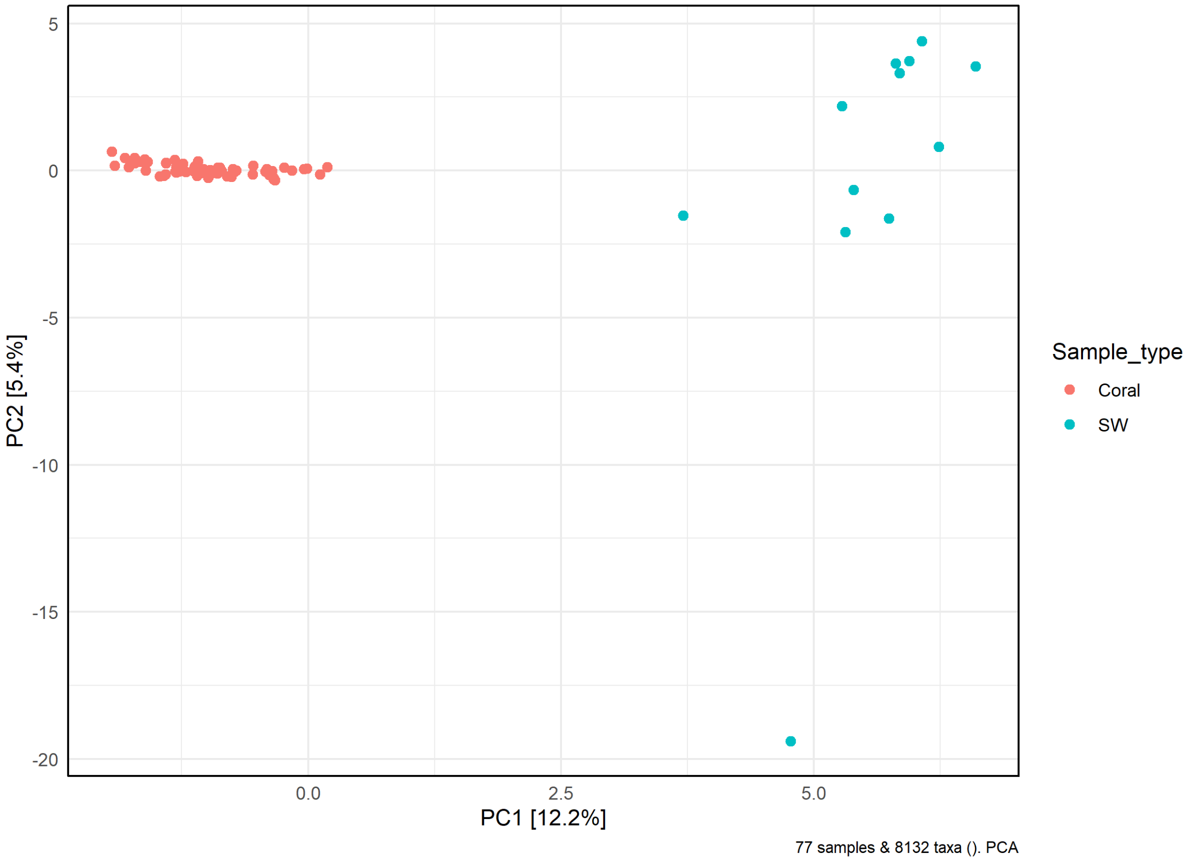

Figure S5: Beta diversity of the microeukaryote community of *C. rubrum* ("Coral", in red) and the seawater samples ("SW", in blue). Principal component analysis based on the Aitchison distance matrix showing the distribution and dispersion of the samples.

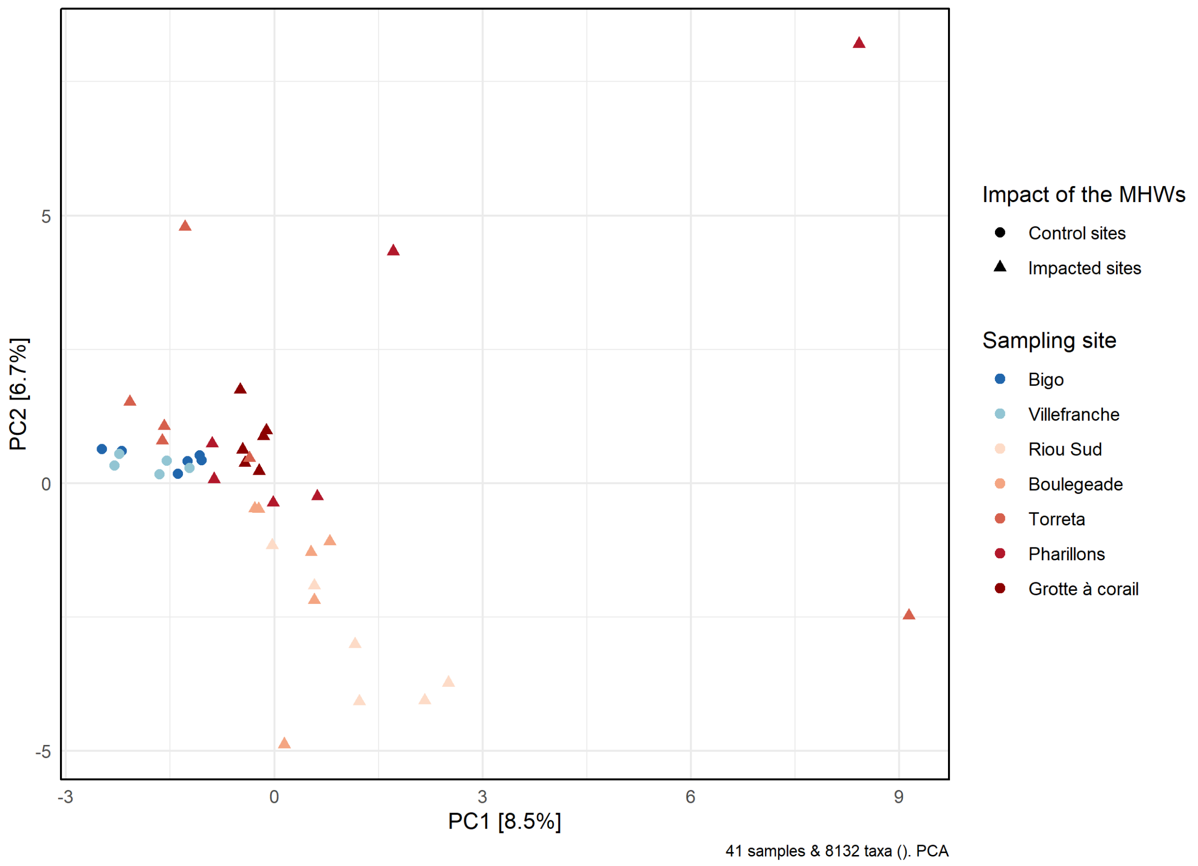

Figure S6: Principal component analysis of the Aitchison distance matrix based on the composition of the microeukaryote community (ASV level) associated with *C. rubrum* samples according to the sampling site and the impact of the MHWs.

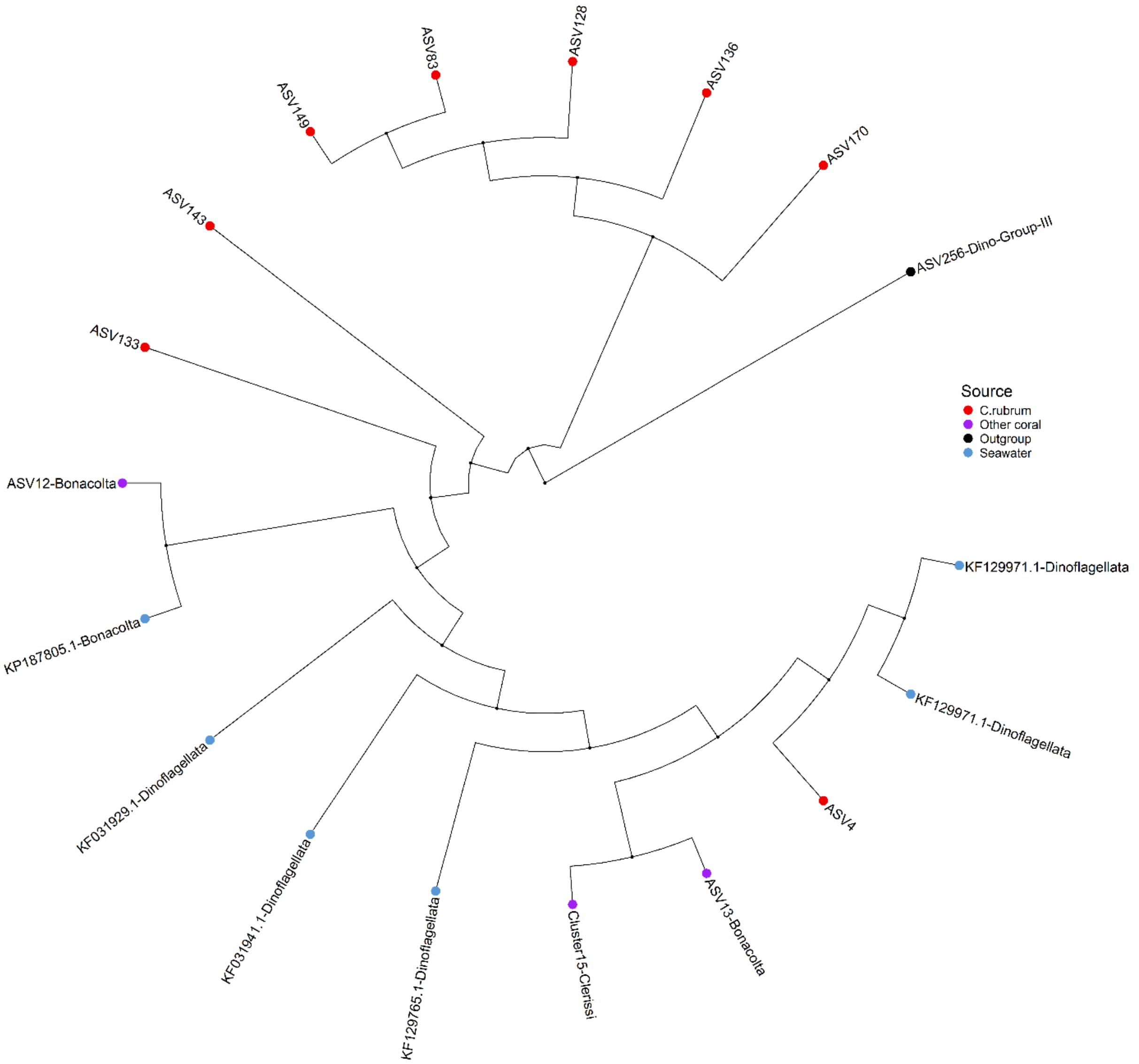

Figure S7: Dendrogram illustrating the phylogenetic relationships (PhyML, GTR model) between all the ASVs annotated as belonging to the Dino Group I Clade 1 family, public ASV sequences recovered after a BLASTn search on the NCBI nucleotide collection using the ASV4 (as the ASV4 is the most prevalent ASV annotated as belonging to the Dino Group I Clade 1 family) as well as ASV sequences recovered from two studies and annotated as belonging to the Dino Group I Clade 1 family (Sequences recovered from *Paramuricea clavata* in Bonacolta et al., 2024 and from *Pocillopora damicornis* in Clerissi et al., 2018).

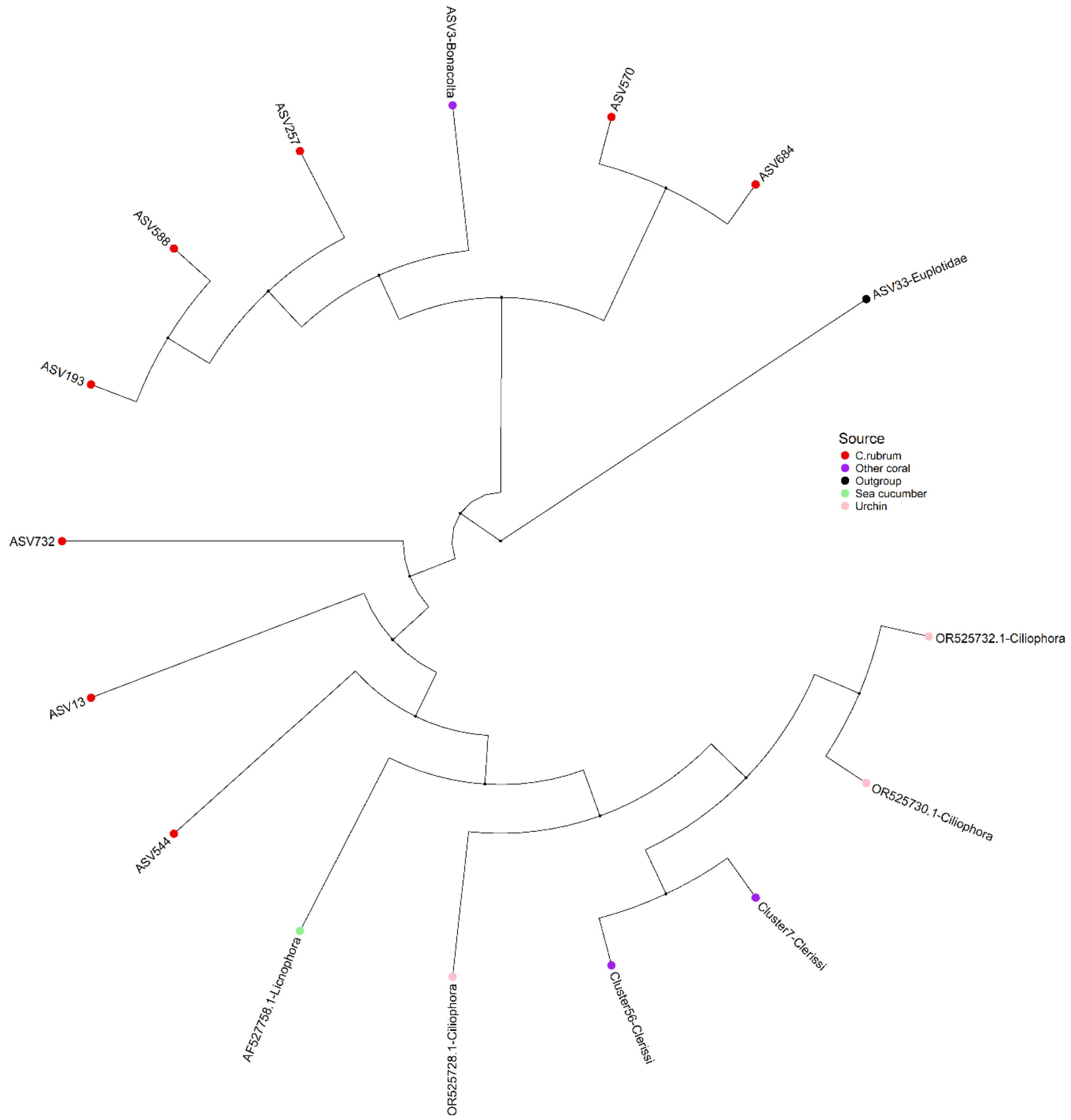

Figure S8: Dendrogram illustrating the phylogenetic relationships (PhyML, TN93 model) between all the ASVs annotated as belonging to the Licnophoridae family, public ASV sequences recovered after a BLASTn search on the NCBI nucleotide collection using the ASV13 (as the ASV13 is the most prevalent ASV annotated as belonging to the Licnophoridae family) as well as ASV sequences recovered from two studies and annotated as belonging to the Licnophoridae family (Sequences recovered from *Paramuricea clavata* in Bonacolta et al., 2024 and from *Pocillopora damicornis* in Clerissi et al., 2018).

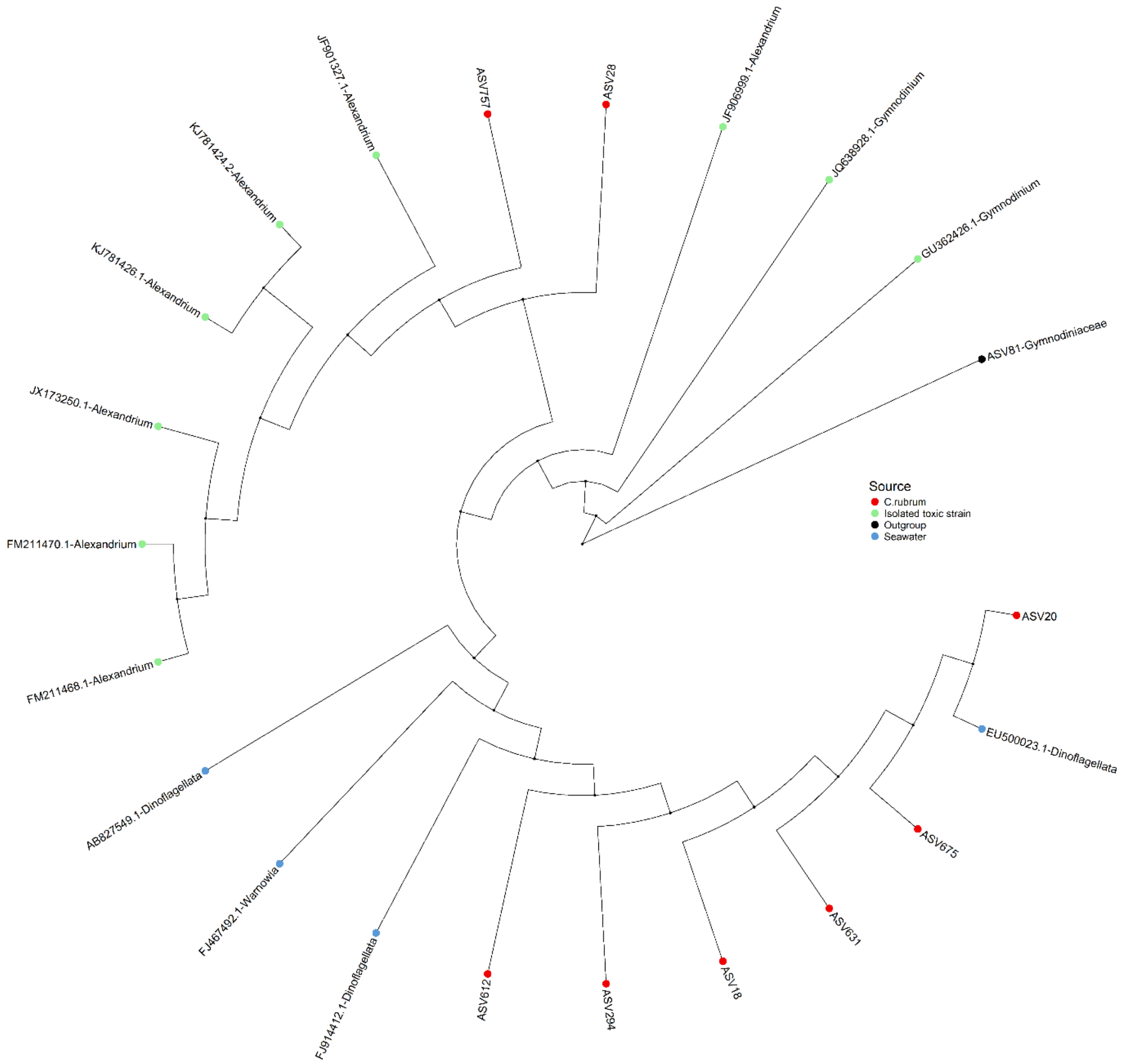

Figure S9: Dendrogram illustrating the phylogenetic relationships (PhyML, GTR model) between all the ASVs annotated as belonging to the Warnowiaceae family, public ASV sequences recovered after a BLASTn search on the NCBI nucleotide collection using the ASV4 (as the ASV4 is the most prevalent ASV annotated as belonging to the Warnowiaceae family).

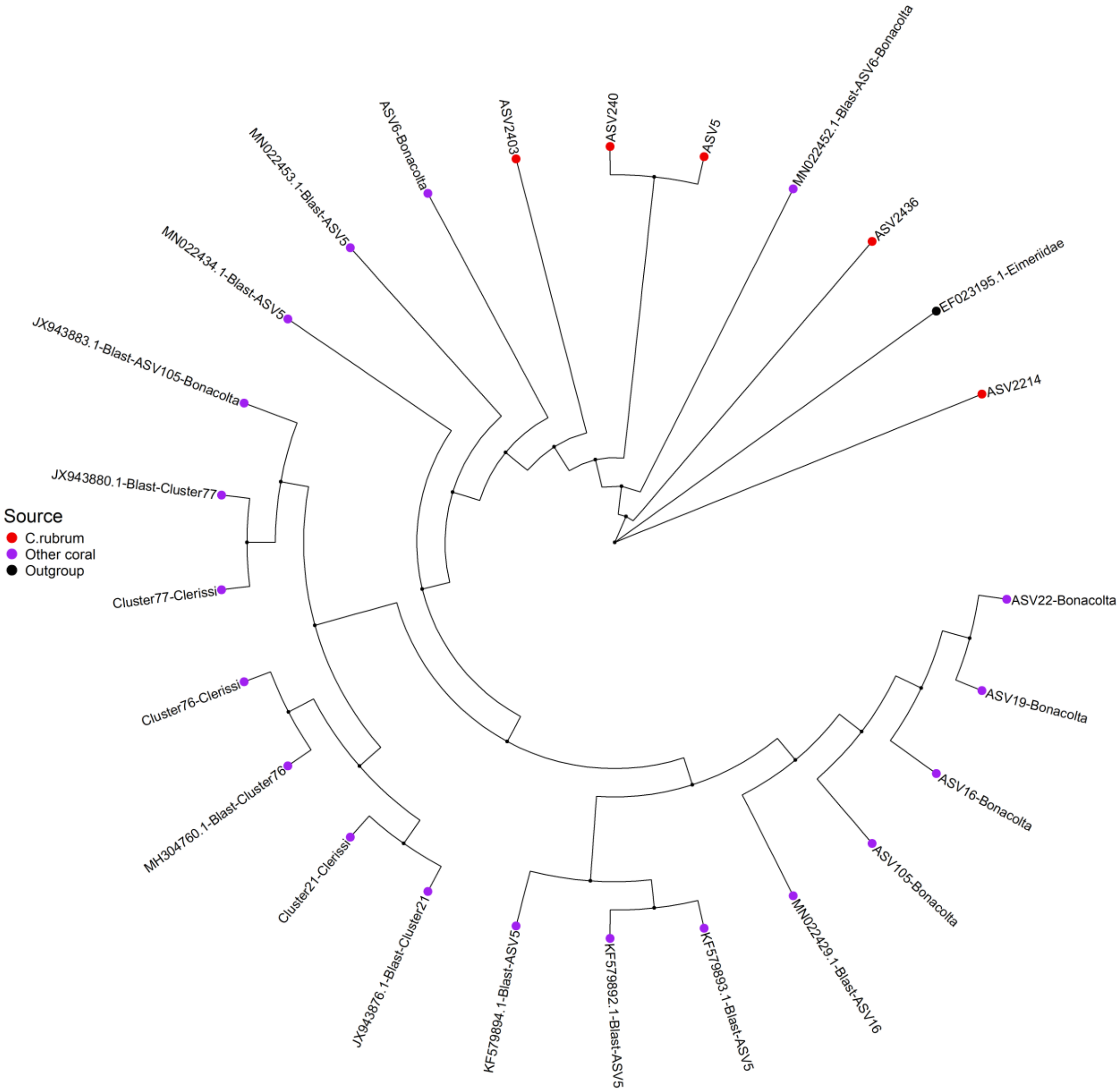

Figure S10: Dendrogram illustrating the phylogenetic relationships (PhyML, TN93 model) between all the ASVs annotated as belonging to the Corallicolidae family, public ASV sequences recovered after a BLASTn search on the NCBI nucleotide collection using the ASV5 (as the ASV5 is the most prevalent ASV annotated as belonging to the Corallicolidae family) as well as ASV sequences recovered from two studies and annotated as belonging to the Corallicolidae family (Sequences recovered from *Paramuricea clavata* in Bonacolta et al., 2024 and from *Pocillopora damicornis* in Clerissi et al., 2018).

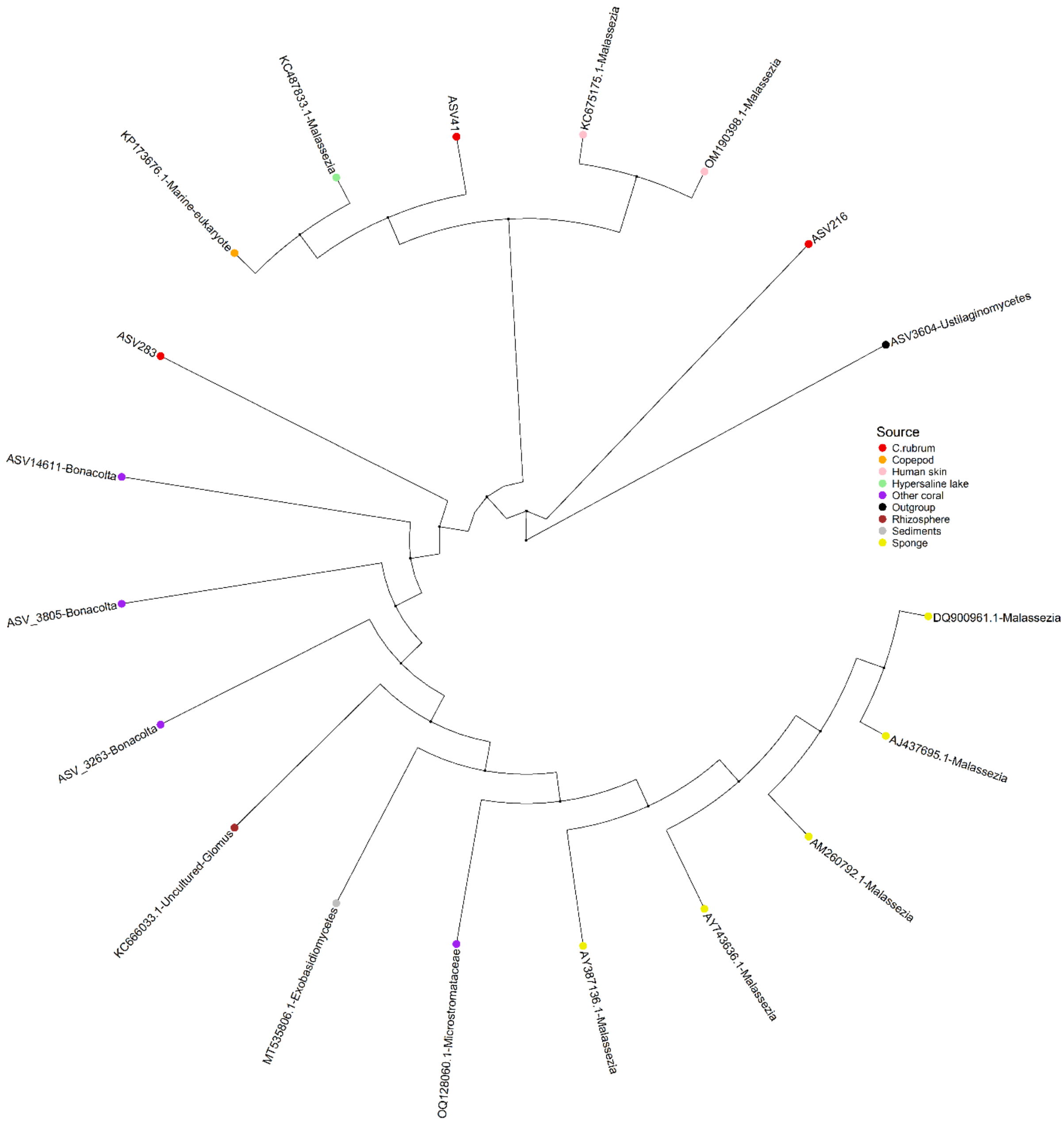

Figure S11: Dendrogram illustrating the phylogenetic relationships (PhyML, TN93 model) between all the ASVs annotated as belonging to the Exobasidiomycetes family, public ASV sequences recovered after a BLASTn search on the NCBI nucleotide collection using the ASV41 (as the ASV41 is the most prevalent ASV annotated as belonging to the Exobasidiomycetes family) as well as ASV sequences recovered from two studies and annotated as belonging to the Exobasidiomycetes family (Sequences recovered from *Paramuricea clavata* in Bonacolta et al., 2024 and from *Pocillopora damicornis, Pavona decussata* and *Porites lutea* in Papan et al., 2023).

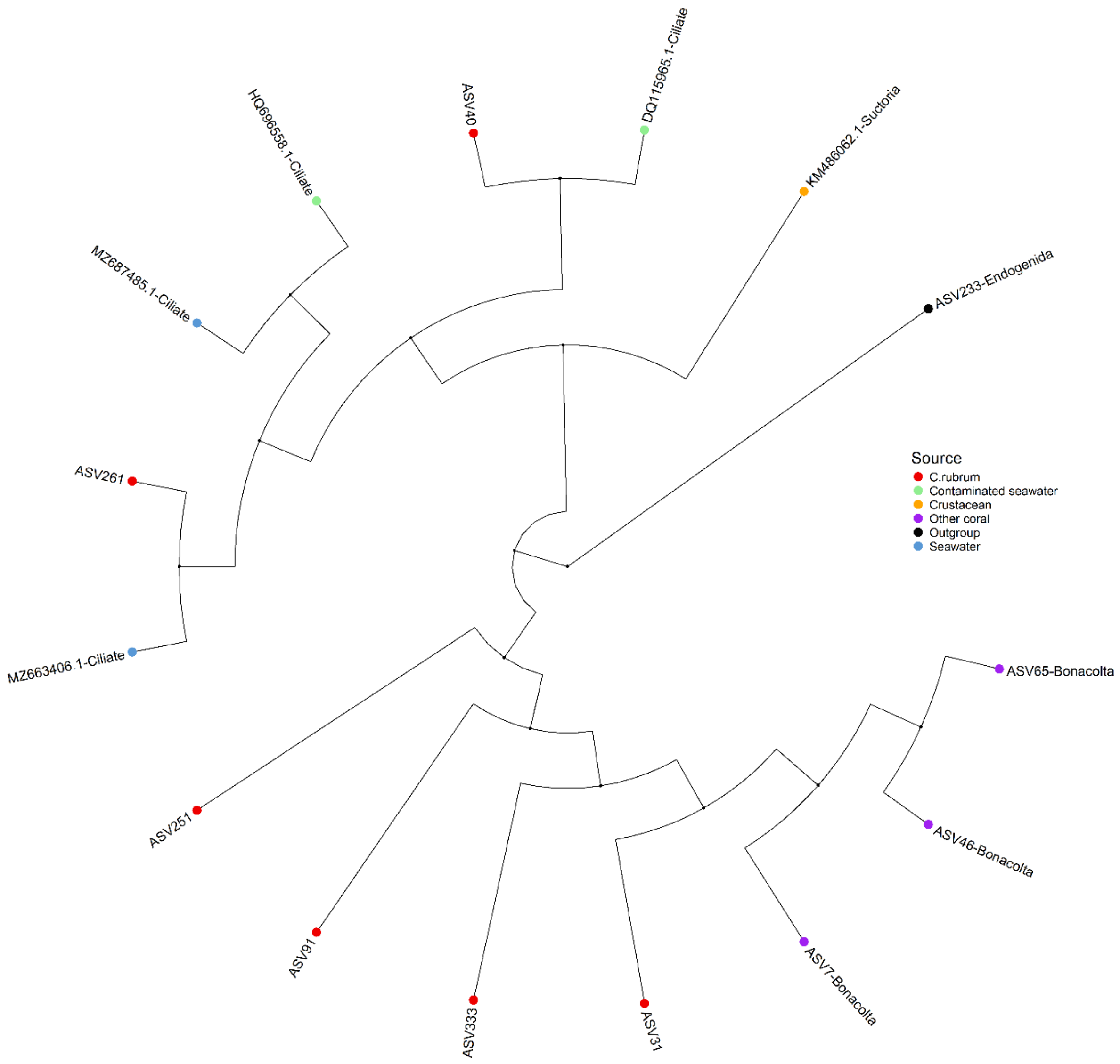

Figure S12: Dendrogram illustrating the phylogenetic relationships (PhyML, GTR model) between all the ASVs annotated as belonging to the Ephelotidae family, public ASV sequences recovered after a BLASTn search on the NCBI nucleotide collection using the ASV40 (as the ASV40 is the most prevalent ASV annotated as belonging to the Ephelotidae family) as well as ASV sequences recovered from one study and annotated as belonging to the Ephelotidae family (Sequences recovered from *Paramuricea clavata* in Bonacolta et al., 2024).

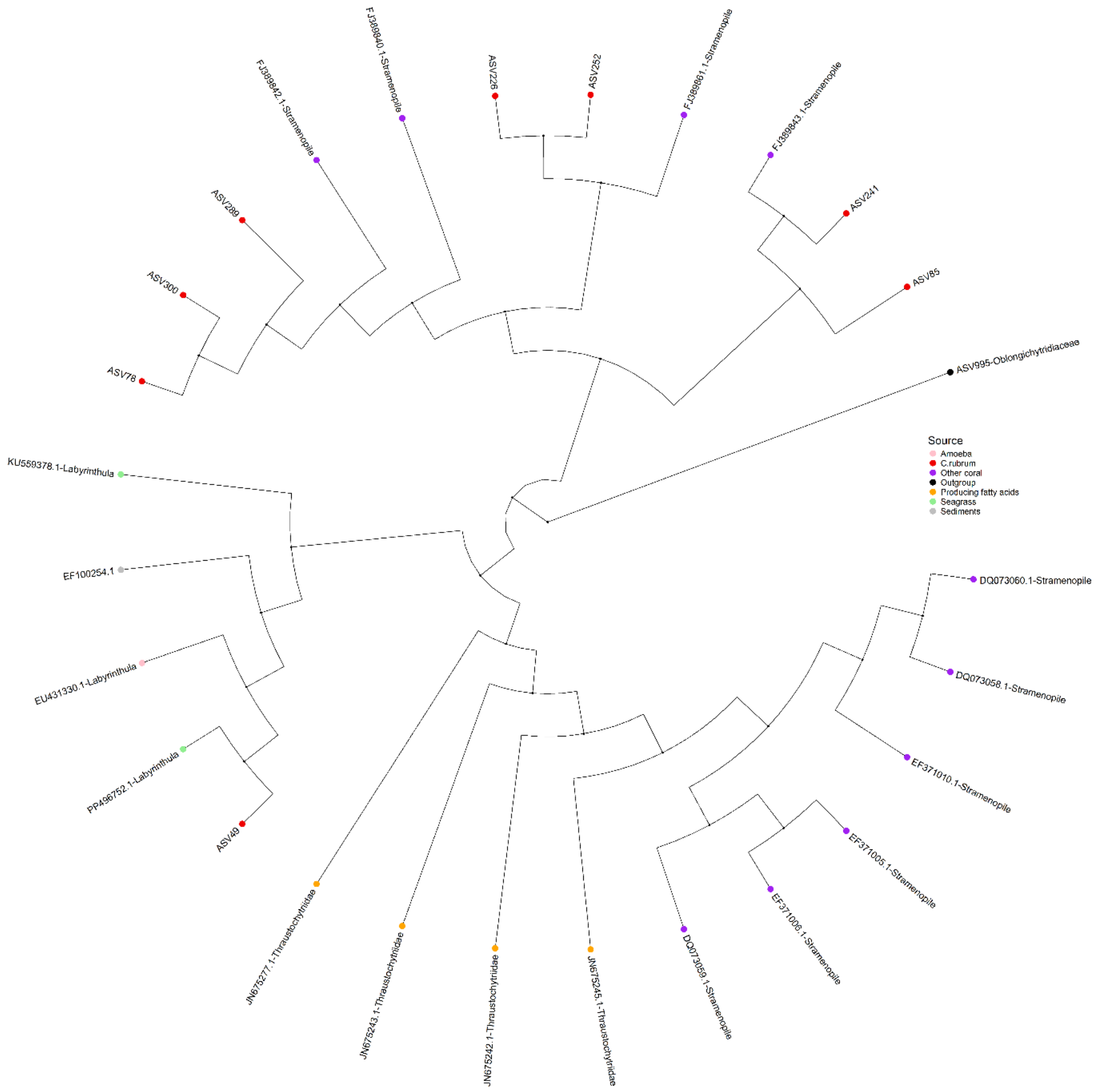

Figure S13: Dendrogram illustrating the phylogenetic relationships (PhyML, HKY85 model) between all the ASVs annotated as belonging to the Labyrinthulaceae family, public ASV sequences recovered after a BLASTn search on the NCBI nucleotide collection using the ASV49 (as the ASV49 is the most prevalent ASV annotated as belonging to the Labyrinthulaceae family) as well as ASV sequences recovered from three studies and annotated as belonging to the Labyrinthulaceae family (Sequences recovered from *Favia* spp. in Siboni et al. 2010, from fungiid corals in Ben-Dov et al. 2009 and from *Fungia granulosa* in Kramarsky-Winter et al. 2006).

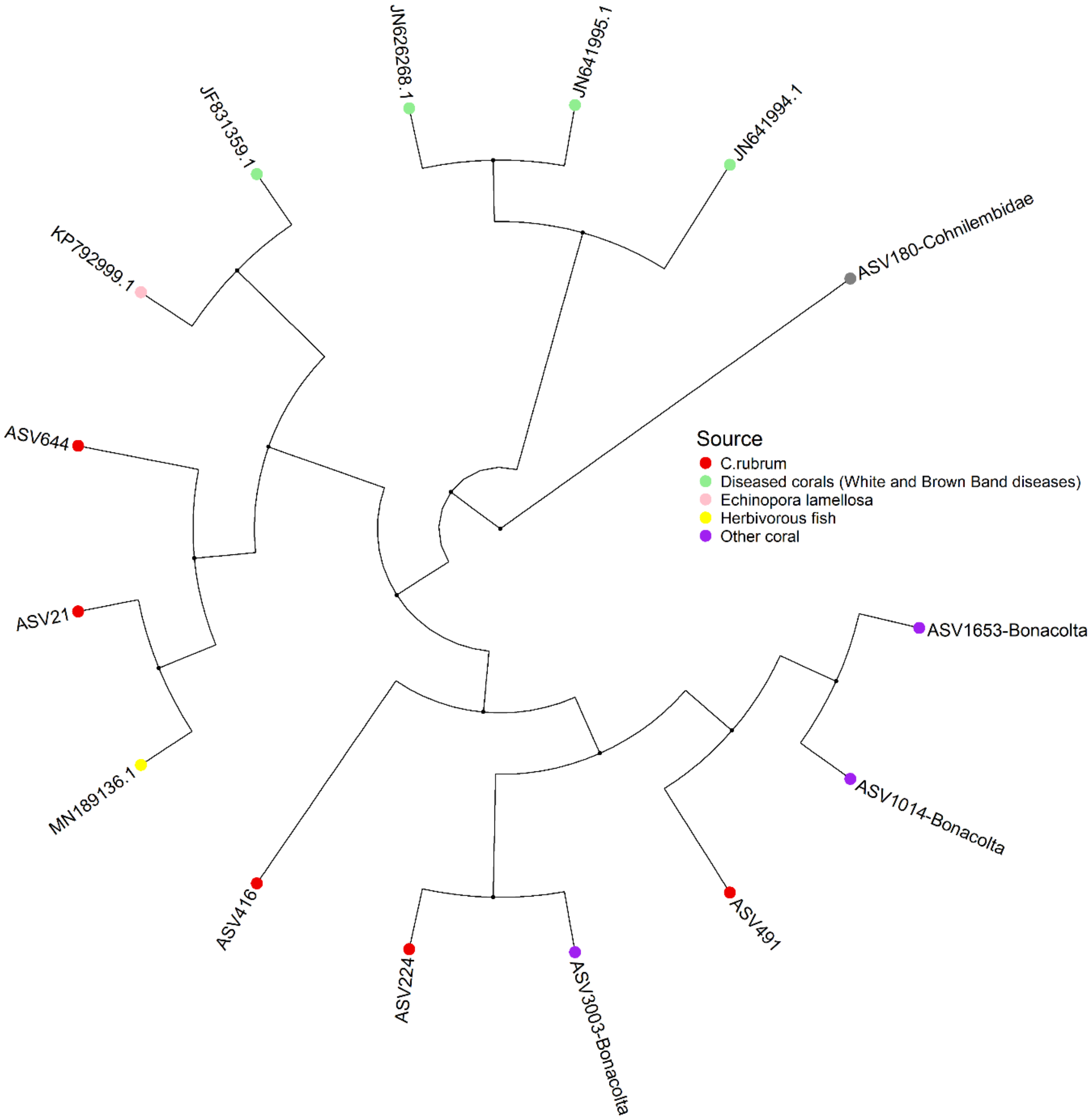

Figure S14: Dendrogram illustrating the phylogenetic relationships (PhyML, GTR model) between all the ASVs annotated as belonging to the Philasterida family, public ASV sequences recovered after a BLASTn search on the NCBI nucleotide collection using the ASV21 (as the ASV21 is the most prevalent ASV annotated as belonging to the Philasterida family) as well as ASV sequences recovered from one study and annotated as belonging to the Philasterida family (Sequences recovered from *Paramuricea clavata* in Bonacolta et al., 2024).
